# Meta-analysis for milk fat and protein percentage using imputed sequence variant genotypes in 94,321 cattle from eight cattle breeds

**DOI:** 10.1186/s12711-020-00556-4

**Published:** 2020-07-07

**Authors:** Irene van den Berg, Ruidong Xiang, Janez Jenko, Hubert Pausch, Mekki Boussaha, Chris Schrooten, Thierry Tribout, Arne B. Gjuvsland, Didier Boichard, Øyvind Nordbø, Marie-Pierre Sanchez, Mike E. Goddard

**Affiliations:** 1 Agriculture Victoria Research, AgriBio, 5 Ring Road, Bundoora, VIC 3083 Australia; 2 Faculty of Veterinary & Agricultural Science, University of Melbourne, Parkville, VIC 3010 Australia; 3 GENO SA, Storhamargata 44, 2317 Hamar, Norway; 4 Animal Genomics, ETH Zurich, Zurich, Switzerland; 5 Université Paris-Saclay, INRAE, AgroParisTech, GABI, 78350 Jouy-en-Josas, France; 6 CRV, PO Box 454, 6800 AL Arnhem, The Netherlands

## Abstract

**Background:**

Sequence-based genome-wide association studies (GWAS) provide high statistical power to identify candidate causal mutations when a large number of individuals with both sequence variant genotypes and phenotypes is available. A meta-analysis combines summary statistics from multiple GWAS and increases the power to detect trait-associated variants without requiring access to data at the individual level of the GWAS mapping cohorts. Because linkage disequilibrium between adjacent markers is conserved only over short distances across breeds, a multi-breed meta-analysis can improve mapping precision.

**Results:**

To maximise the power to identify quantitative trait loci (QTL), we combined the results of nine within-population GWAS that used imputed sequence variant genotypes of 94,321 cattle from eight breeds, to perform a large-scale meta-analysis for fat and protein percentage in cattle. The meta-analysis detected (p ≤ 10^−8^) 138 QTL for fat percentage and 176 QTL for protein percentage. This was more than the number of QTL detected in all within-population GWAS together (124 QTL for fat percentage and 104 QTL for protein percentage). Among all the lead variants, 100 QTL for fat percentage and 114 QTL for protein percentage had the same direction of effect in all within-population GWAS. This indicates either persistence of the linkage phase between the causal variant and the lead variant across breeds or that some of the lead variants might indeed be causal or tightly linked with causal variants. The percentage of intergenic variants was substantially lower for significant variants than for non-significant variants, and significant variants had mostly moderate to high minor allele frequencies. Significant variants were also clustered in genes that are known to be relevant for fat and protein percentages in milk.

**Conclusions:**

Our study identified a large number of QTL associated with fat and protein percentage in dairy cattle. We demonstrated that large-scale multi-breed meta-analysis reveals more QTL at the nucleotide resolution than within-population GWAS. Significant variants were more often located in genic regions than non-significant variants and a large part of them was located in potentially regulatory regions.

## Background

The identification of causal mutations is important to take full advantage of sequence data to improve the accuracy of genomic prediction [1, 2]. Furthermore, it contributes to a better understanding of the biological mechanisms that underlie variation in quantitative traits and diseases. Since an increasing amount of sequence data is becoming available, it is possible to perform sequence-based genome-wide association studies (GWAS) to identify candidate causal mutations or markers in high linkage disequilibrium (LD) with them. However, due to the stringent thresholds that are necessary to avoid false positive associations, very large cohorts with both sequence variant genotypes and phenotypes are required to identify quantitative trait loci (QTL) with small to moderate effects.

Increasing the statistical power to identify candidate causal variants is possible by combining multiple datasets. However, in practice, the sharing of data at the individual level is not always possible, and prevents the compilation of large mapping cohorts. An alternative approach to compiling large mapping cohorts without exchanging data at the individual level is to perform a meta-analysis that uses GWAS summary statistics to approximate a GWAS using the full, combined dataset [3, 4]. For stature, an international collaboration has shown that a large-scale, across-population meta-analysis provides high power to detect trait-associated variants [5]. Recently, several other meta-analyses have been carried out in cattle for multiple traits including fat and protein percentages in milk [6–9]. To date, the largest meta-analysis for fat and protein percentage included the single nucleotide polymorphism (SNP) chip genotypes of 78,772 cows from three dairy cattle breeds in France [9]. Performing a meta-analysis of summary statistics generated from multiple breeds across multiple countries also facilitates the combination of data from mapping cohorts that do not contain the same set of variants or that were prepared using different imputation or association methods.

In addition to achieving sufficient power to identify trait-associated variants, high precision is important in GWAS in order to prioritize a small number of variants as candidate causal mutations. Because of the long-range LD that is present in most cattle breeds [10], many variants in high LD tag the same QTL, which makes the identification of causal variants a difficult task. Because LD is conserved over shorter distances across breeds than within a breed, a multi-breed GWAS or across-breed meta-analysis can improve mapping precision [6].

Although direct selection on milk composition in dairy cattle has been more limited than selection on milk yield, a correlated response would be expected due to the genetic correlation between milk yield and composition. While all dairy breeds have been selected for milk yield and hence composition, slight differences in selection pressures may have occurred in different breeds. Pausch et al. [7] reported higher F_ST_ values for QTL than non-QTL, suggesting differences in selection pressures in the breeds used in their study.

To maximise both power and precision of a GWAS for fat and protein percentages in milk, we carried out meta-analyses of the summary statistics of nine within-population GWAS that used imputed sequence variant genotypes of 94,321 individuals representing eight cattle breeds. Besides identifying QTL for each trait, significant and non-significant variants were compared in terms of minor allele frequency (MAF), functional annotations and F_ST_.

## Methods

As input for the meta-analyses, summary statistics of nine within-population GWAS were used. In total, the GWAS included imputed sequence variant genotypes of 94,321 individuals. The within-population GWAS are summarized in Table 1.

**Table 1:** Description of GWAS used in the meta-analysis

Acronym	Country	Breeds	Sex	Pheno	GWAS	impRef	impSoft	nIds	nVar
AUSB	Australia, New Zealand, the Netherlands	Holstein, Jersey, Australian Red	Bulls	DYD	GCTA	1000_Run6	Minimac3	11,923	15,474,359
AUSC	Australia	Holstein, Jersey, Australian Red	Cows	YD	GCTA	1000_Run6	Minimac3	32,347	15,400,322
HOLF	France	Holstein	Bulls	DYD	GCTA	1000_Run4	FImpute	6375	13,885,363
MON	France	Montbéliarde	Bulls	DYD	GCTA	1000_Run4	FImpute	2588	14,409,070
NOR	France	Normande	Bulls	DYD	GCTA	1000_Run4	FImpute	2319	13,937,693
NR	Norway	Norwegian Red	Bulls, cows	(D)YD	GCTA	within breed	Minimac4	21,540	12,985,160
HOLG	Germany	Holstein	Bulls	EBV	EMMAX	1000_Run4	Minimac3	8805	14,804,061
BRAU	Switzerland	Braunvieh	Bulls	EBV	EMMAX	1000_Run5	Minimac3	1646	15,813,995
FLCK	Germany/Austria	Fleckvieh	Bulls	DYD	EMMAX	1000_Run5	Minimac3	6778	17,042,717
Total								94,321	25,702,992

Pheno: phenotypes used i.e. daughter yield deviations (DYD, bulls), yield deviations (YD, cows), estimated breeding values (EBV), GWAS: software used for GWAS, impRef: imputation reference, impSoft: imputation software; nIds: number of individuals; nVar: number of variants; nIds and nVar were the same except in Norwegian Red (21,550 individuals and 12,985,177 variants for protein content)

### Phenotypes used for within-population GWAS

Phenotypes were either yield deviations (YD) of cows, i.e. own mean performances adjusted for environmental effects, or daughter yield deviations (DYD) of bulls, i.e. average daughter performance adjusted for environmental effects and for breeding value of the mates, or a combination of those. Two studies simply used estimated breeding values (EBV) of bulls, which were not deregressed. However, considering the high relatability of the traits (0.89 in BSW and 0.95 in HOL [7]), the contribution of information from relatives to the EBV is very small and should not have any major consequences on the GWAS [11].

The GWAS for the Australian dataset were performed across breeds, but separately for bulls (AUSB) and cows (AUSC). The Australian animals and the GWAS model are described in a previous report [12]. Briefly, the AUSB dataset contained 9739 Holstein, 2059 Jersey and 125 Australian Red bulls, and the AUSC dataset consisted of 22,899 Holstein, 6174 Jersey, 424 Australian Red and 2850 crossbred cows. Phenotype data included 6569 CRV bulls (https://www.crv4all-international.com/) with phenotypes derived from their Interbull MACE breeding values (https://interbull.org/ib/interbullactivities), deregressed to the Australian scale, and converted to the scale of the daughter trait deviation. The remaining 5354 bulls and all 32,347 cows were from DataGene (https://datagene.com.au/). The GWAS for the Norwegian population (Norwegian Red cattle, NR) was performed using data on 21,540 and 21,550 bulls and cows, for fat and protein percentage, respectively. All other GWAS were performed within breed and sex. More details on the HOLG, BRAU and FLCK GWAS can be found in [7].

### Genotypes used for within-population GWAS

Only variants with a MAF lower than 0.002 or a minor allele count (MAC) higher than 4 and, if available, an imputation r^2^ (as provided by the imputation program) ≥ 0.4 was considered for the within-population GWAS. In total, 25,702,992 (25,702,995) distinct variants were analysed for fat (protein) percentage, with the number of variants per within-population GWAS ranging from 12,985,160 to 17,042,717. In total, 7,520,048 (7,520,050) variants were common to all GWAS for fat (protein) percentage.

Most GWAS populations were imputed using multi breed reference populations that comprised 1147, 1557 or 2333 individuals from Run 4, 5 or 6 of the 1000 bulls genomes project, respectively [13], except for the Norwegian Red population, that was imputed using a within-breed reference population of 378 Norwegian Red bulls. Imputation was done using Minimac3 [14], Minimac4 [14] and FImpute [15].

### Within-population GWAS

GWAS were carried out by single SNP regressions using best-guess genotypes and the mixed linear model association (MLMA) analysis as implemented in the GCTA software [16], or using imputed allele dosages and the MLMA approach as implemented in the EMMAX software [17].

### Meta-analysis

All 25,702,992 variants that were present in at least one of the within-population GWAS were included in the meta-analysis. The meta-analysis was based on the weighted Z-scores model as implemented in the METAL software [18] that considers the p-value, direction of effect and number of individuals included in each within-population GWAS. Because the scaling of the phenotypes used for the within-population GWAS differed between the populations, we used the weighted Z-scores model that uses the significance and direction of marker effects as input, rather than alternative models that use allele substitution effects and corresponding standard errors. Van den Berg et al. [6] found that, when combining GWAS with summary statistics from multiple GWAS with difference in scaling of the phenotypes, the weighted Z-scores model yielded results that were very similar to those obtained by a full analysis combining all data used for the GWAS. For each variant and each within-population GWAS, Z-scores were computed as:

$$Z_{k} = \varPhi^{ - 1} \left( {1 - \frac{{p_{k} }}{2}} \right) \times \Delta_{k} ,$$

where $Z_{k}$ is the Z-score for GWAS $k$, $p_{k}$ the p-value estimated in GWAS $k$, $\Delta_{k}$ the direction of effect in GWAS $k$, and $\varPhi$ and $\varPhi^{ - 1}$ are the standard normal cumulative distribution function and its inverse, respectively. Subsequently, overall Z-scores were computed as:

$$Z = \frac{{\mathop \sum \nolimits_{k} z_{k} w_{k} }}{{\sqrt {\mathop \sum \nolimits_{k} w_{k}^{2} } }},$$

where $w_{k}$ is the square root of the number of individuals used in GWAS $k$. An overall p-value was then computed as:

$$p = 2\varPhi \left( { - \left| Z \right|} \right).$$

### QTL detection

All variants with a p-value lower than 10^−8^ were declared significant. To account for multiple testing, the false discovery rate (FDR) was calculated for each within-population GWAS and the meta-analysis as $FDR = \left( {nVariants \times 10^{ - 8} } \right)/nSign$, where $nVariants$ is the number of variants included in the GWAS and $nSign$ is the number of variants with a p-value lower than 10^−8^. QTL were selected by first ordering the significant variants based on their p-values, and subsequently selecting the most significant variants first, with at least 1 Mb between adjacent QTL. Variants within 1 Mb of a more significant variant were assumed to be part of the more significant QTL and not selected as additional QTL.

### COJO

Because LD may be conserved along longer distances than 1 Mb, we performed a conditional and joint analysis (COJO) as implemented in GCTA [19] to test how many of the QTL detected in the meta-analysis appeared to be independent. As a reference sample to estimate the LD structure, we used sequence data of 53 Fleckvieh, 451 Holstein, 90 Jersey, 55 Montbéliarde, 45 Normande and 25 Norwegian Red individuals that were included in Run 6 of the 1000 Bulls genome project [13]. We only included the top variants selected as QTL in the COJO analysis and set the window size to 100 Mb.

### Validation meta-analysis

To validate the QTL detected in the meta-analysis, we performed a second meta-analysis using data on 34,860 cows not included in the original meta-analysis. These cows originated from two countries, Australia and France, and four breeds, Holstein, Jersey, Montbéliarde and Normande. The French populations used in the validation study are described in more detail by Sanchez et al. [20]. Table 2 summarizes the four within-population GWAS that were used as input for the validation meta-analysis. The validation meta-analysis was performed only for the QTL that were detected in the first meta-analysis and that segregated in at least one of the four validation populations. These within-population GWAS and the validation meta-analysis were performed in the same manner as described above for the original analysis. To validate QTL, we compared the direction of the Z-score and p-value in the meta-analyses. Our previous study showed that the comparison of the direction of the effect of variants across different GWAS results can be more powerful in detecting consistent signals than the sole comparison of p-values between different GWAS [12].

**Table 2:** Description of the GWAS used in the validation meta-analysis

Acronym	Country	Breeds	Sex	Pheno	GWAS	impRef	impSoft	nIds
VAUSC	Australia	Holstein, Jersey	Cows	YD	GCTA	1000_Run6	Minimac3	26,953
VHOLF	France	Holstein	Cows	YD	GCTA	1000_Run4	FImpute	2216
VMON	France	Montbéliarde	Cows	YD	GCTA	1000_Run4	FImpute	3032
VNOR	France	Normande	Cows	YD	GCTA	1000_Run4	FImpute	2659
Total								34,860

Pheno: phenotypes used were yield deviations (YD, cows); GWAS: software used for GWAS; impRef: imputation reference; impSoft: imputation software; nIds: number of individuals

### Minor allele frequencies

To compare the MAF of significant variants with the MAF of all variants, we estimated the allele frequencies of the total population used for the meta-analyses. First, the allele counts at each position were computed using the allele frequency in each population. Then, the allele counts were combined and used to estimate the MAF of each variant in the whole population used for the meta-analyses.

### Functional annotations

Functional annotations were compared between significant and all other variants in order to determine if certain functional categories were enriched for trait-associated variants. Genomic coordinates and functional annotations were obtained according to the UMD3.1 assembly of the bovine genome and Ensembl’s Variant Effect Predictor [21, 22]. We used LiftOver (https://genome.ucsc.edu/cgi-bin/hgLiftOver) to convert the positions of the detected QTL from UMD3.1 to their positions on the new ARS-UCD1.2 genome.

### eQTL analysis

Results of the meta-analyses were compared with those of a previous eQTL study [23, 24] to identify potential overlap between QTL and eQTL. The eQTL study contained data of 105 Holstein and 26 Jersey cows. In total, 9,191,239 and 8,587,100 variants were included in both the eQTL study using white blood cells and cells collected from milk samples, respectively, and the meta-analysis. The cells collected from milk samples included immune cells and mammary gland epithelial cells. The transcriptome of cells collected from milk samples shared a high similarity with that of the mammary gland tissue. A detailed description of the RNA sequence data generation for each tissue is reported in [25]. The association between the variants and gene expression was estimated using a linear model. A variant was declared as an eQTL if its genotype was significantly associated with the expression of a gene located within 1 Mb of the variant with a p-value ≤ 10^−6^.

### F_ST_

To investigate whether significant SNPs are associated with higher F_ST_ values and investigate the potential presence of different selection pressures between breeds, we calculated F_ST_ values for 16,626,224 sequence SNPs using allele frequencies in 53 Fleckvieh, 451 Holstein, 90 Jersey, 55 Montbéliarde, 45 Normande and 25 Norwegian Red individuals that were included in Run 6 of the 1000 Bulls genome project [13]. F_ST_ values were computed for all breeds combined according to Weir and Cockerham [26], as implemented in GCTA [16], to measure the divergence between the breeds in the meta-analysis.

### DAVID analysis

We used the Database for Annotation, Visualization and Integrated Discovery (DAVID) functional annotation tool [27, 28] to investigate if gene ontology terms were enriched for genes located within the QTL. Genes with variants that were significant in the meta-analysis and located in or near the gene, according to previously described annotation, were used as input for DAVID. In the DAVID analysis, we included the following terms: COG_ONTOLOGY, UP_KEYWORDS, UP_SEQ_FEATURE, GOTERM_BP_DIRECT, GOTRM_CC_DIRECT, GOTERM_MF_DIRECT, KEGG_PATHWAY and UP_TISSUE.

## Results

### Number of QTL detected

**Fig. 1:**
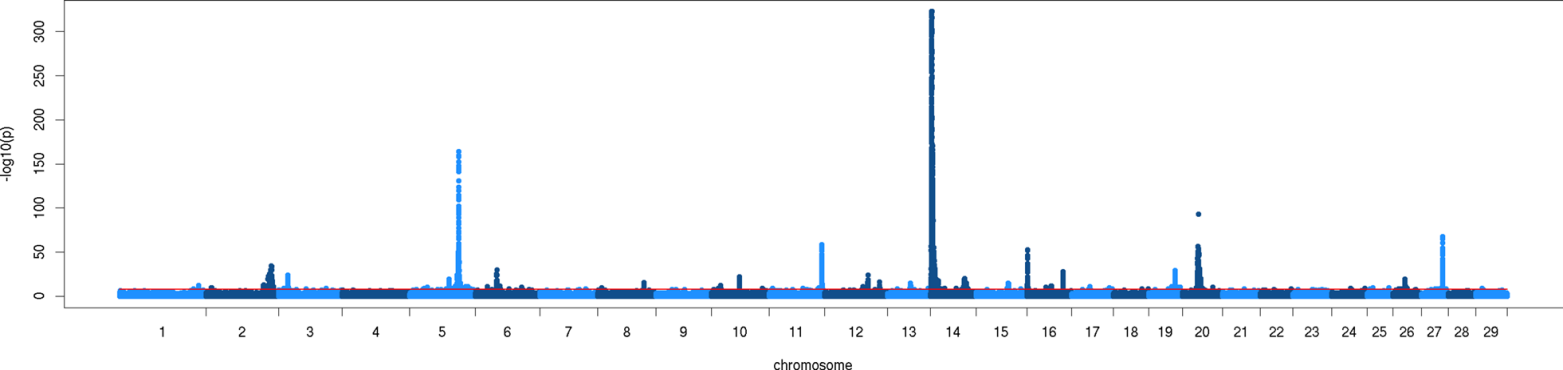
Manhattan plot of the meta-analysis for fat percentage. Red line indicates p = 10^−8^

**Fig. 2:**
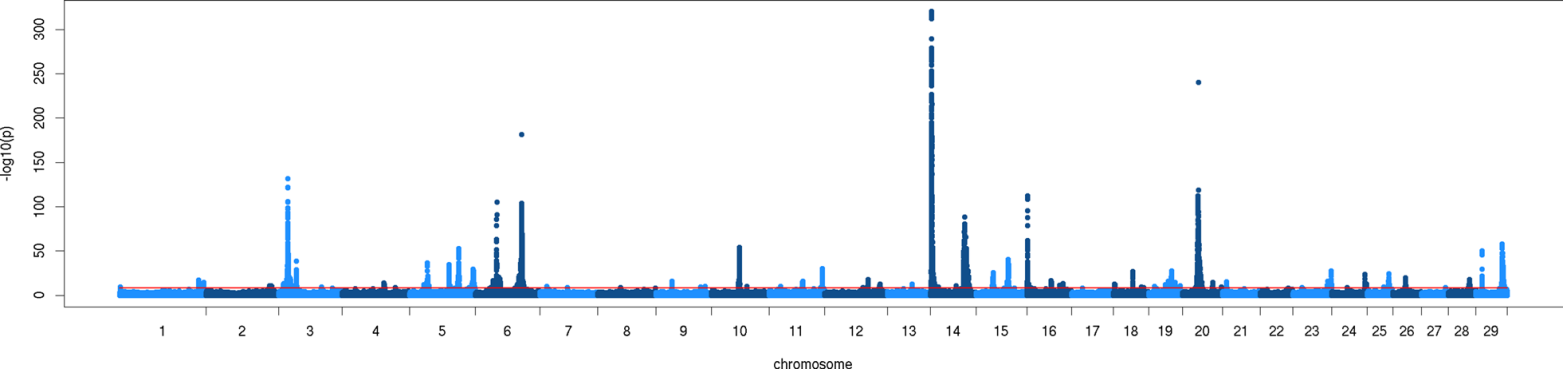
Manhattan plot of the meta-analysis for protein percentage. Red line indicates p = 10^−8^

Figures 1 and 2 show Manhattan plots of the meta-analysis for fat and protein percentage, respectively. Manhattan plots of the within-population GWAS are in Additional file 1: Figure S1. Table 3 compares the number of significant variants and QTL in the within-population GWAS and the meta-analysis. The number of significant variants detected in the within-population GWAS ranged from 2117 for protein percentage in BRAU to 13,955 for fat percentage in AUSB. For protein percentage, the meta-analysis detected more variants than all the GWAS combined together, while for fat percentage, all the GWAS combined together detected more significant variants than the meta-analysis.

**Table 3:** Number of variants and QTL detected in the GWAS and meta-analysis for fat and protein percentage

Analysis	Fat %				prot %			
	nS	FDR	nQ	nS/nQ	nS	FDR	nQ	nS/nQ
AUSB	8871	1.7 × 10^−5^	56	158	13,955	1.1 × 10^−5^	52	268
AUSC	9502	1.6 × 10^−5^	74	128	13,475	1.1 × 10^−5^	49	275
HOLF	10,124	1.4 × 10^−5^	22	460	11,033	1.3 × 10^−5^	35	315
MON	3971	3.6 × 10^−5^	13	305	5383	2.7 × 10^−5^	19	283
NOR	2981	4.7 × 10^−5^	11	271	3379	4.1 × 10^−5^	16	211
NR	4304	3.0 × 10^−5^	30	143	6231	2.1 × 10^−5^	37	168
HOLG	9244	1.6 × 10^−5^	20	462	10,102	1.5 × 10^−5^	38	266
BRAU	2483	6.4 × 10^−5^	13	191	2117	7.5 × 10^−5^	9	235
FLCK	9492	1.8 × 10^−5^	20	475	5654	3.0 × 10^−5^	33	171
GWAS	31,559	–	124	255	42,518	–	104	409
META	27,820	9.2 × 10^−6^	138	202	44,095	5.8 × 10^−6^	176	251

fat %: fat percentage; prot %: protein percentage; nS: number of significant variants; FDR: false discovery rate; nQ: number of QTL; nS/nQ: number of significant variants per QTL; AUSB: Australian bull dataset; AUSC: Australian cow dataset; HOLF: French Holstein; MON: Montbéliarde; NOR: Normande; NR: Norwegian Red; HOLG: German Holstein; BRAU: Braunvieh; FLCK: Fleckvieh; GWAS: non-overlapping significant variants select in any of the 9 GWAS; META: meta-analysis

For both fat and protein percentage, more QTL were detected in the meta-analysis than with the within-population GWAS. The lists of the QTL detected in the meta-analysis are in Additional file 2: Tables S1 and Additional file 3: Table S2.

Several of the QTL detected in the meta-analysis were not significant in any of the within-population GWAS. For example, the meta-analysis detected a QTL for protein percentage located at 7,924,949 bp on chromosome 3, that had a p-value of 9.4 × 10^−14^ in the meta-analysis. In the within-population GWAS, the highest significance for this variant was for the NR breed with a p-value of 8.4 × 10^−4^.

Multiple variants were significant in the within-population GWAS, but not in the meta-analysis. Most of these variants had inconsistencies in direction of effect between populations in the within-population GWAS. For example, a QTL for protein percentage was detected in the NR breed (p-value of 2.7 × 10^−10^) at 35,509,237 bp on chromosome 25 (see Additional file 4: Figure S2). The alternate allele of the lead variant had a positive effect in the AUSB, AUSC, HOLF, MON, NOR, and FLCK breeds, but a negative effect in the NR, HOLG and BRAU breeds, and was not significant in any GWAS except in the GWAS for NR. The meta-analysis revealed a QTL nearby at 36,527,270 bp that was only included in the GWAS for NR and had a p-value of 3.7 × 10^−25^. Visual inspection of this region on chromosome 25 indicated that a possible peak visible in the GWAS for NR and the meta-analysis, that encompasses both the QTL at 35.5 Mb and 36.5 Mb, with fewer significant variants associated with the peak in the meta-analysis than the GWAS (see Additional file 4: Figure S2)

### COJO

Out of the 138 and 176 QTL detected for fat and protein percentage, 132 and 159 were present in the dataset that was used to estimate the LD structure for COJO. The COJO analyses retained 74 QTL for fat percentage and 84 QTL for protein percentage with a p-value ≤ 10^−8^. In most cases, the discarded variant was close to another variant that was retained. This implies that the two variants mark only a single QTL and not two independent QTL. Nevertheless, four variants were retained between the start of chromosome 14 and 5 Mb, which implies that there are at least three other QTL for fat percentage in this region as well as *DGAT1*. For each QTL, Additional file 2: Tables S1 and Additional file 3: Table S2 indicate whether QTL were retained by COJO or not, and the p-value in the COJO analysis.

### Validation meta-analysis

Of the 138 QTL detected for fat percentage, 123 were present in the validation analysis, of which 107 (87%) had the same direction of Z-score in the original meta-analysis and the validation analysis. Fifteen of these QTL (12.2%) were significant (p ≤ 10^−8^) in the validation analysis. For protein percentage, 158 of the 176 QTL detected in the meta-analysis were present in the validation analysis. One hundred % of the QTL had the same direction of Z-score and 28 (17.7%) were significant in both analyses. Additional file 2: Table S1 and Additional file 3: Table S2 show for each QTL if they were present in the validation analysis, if they had the same direction of Z-score, and the p-value in the validation analysis.

### Minor allele frequency

**Fig. 3:**
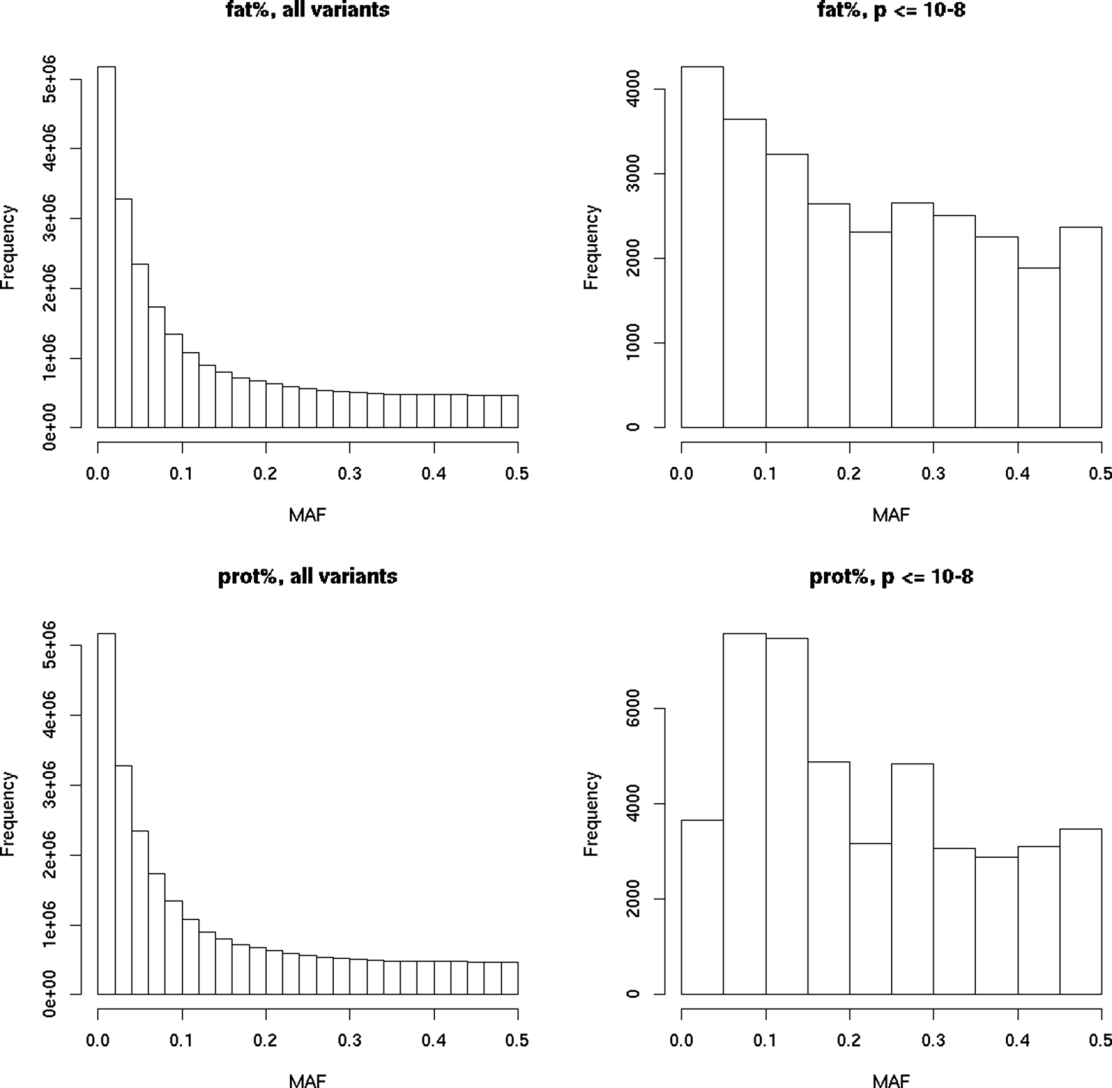
Distribution of minor allele frequencies (MAF) of all variants and significant variants. Significant variants had a p-value ≤ 10^−8^ in the meta-analysis

The sequence variants were enriched for low-frequency MAF classes. However, variants that were significant in the meta-analyses were only slightly enriched for low MAF classes (Fig. 3). For example, 46% of all variants had a MAF between 0.1 and 0.5, whereas this was the case for 72% of the significant variants detected for fat percentage, and 75% of the significant variants detected for protein percentage. A similar pattern was found when comparing the allele frequencies of significant and all variants within a population (see Additional file 5: Figure S3 and Additional file 6: Figure S4). Especially in the Holstein populations, most of the significant QTL had moderate to large MAF.

### Functional annotations

Significant variants in the meta-analysis were more often located in or near genes than all variants (Table 4). Nearly two-thirds (65.85%) of all variants tested were intergenic. For the significant variants, the percentage of intergenic variants was substantially lower, i.e. ~ 50%. All other annotation classes were more frequent for significant than for all variants. For example, the percentage of splice variants was more than twice as high for significant than for all variants (0.14 vs. 0.06%).

**Table 4:** Percentage of variants in functional classes of annotation

Annotation	All variants	p_fat % ≤ 10^−8^	p_prot % ≤ 10^−8^
Intergenic	65.85	51.45	50.07
Intron	26.54	35.12	35.84
Upstream_gene	3.49	5.91	5.89
Downstream_gene	3.04	4.24	4.83
Synonymous	0.36	0.99	0.91
Missense	0.32	0.54	0.54
3_prime_UTR	0.22	0.32	0.44
Splice_region	0.06	0.14	0.14
5_prime_UTR	0.05	0.12	0.11
Non_coding_transcript_exon	0.03	0.15	0.12
Other	0.02	0.01	0.02
Not annotated	0.02	1.01	1.09

p_fat % ≤ 10^−8^ and p_prot % ≤ 10^−8^ = variants with a p-value ≤ 10^−8^ in the meta-analysis for fat and protein percentage, respectively

### eQTL

Table 5 shows the overlap between eQTL and significant variants in the meta-analysis. When blood cells were used in the eQTL analysis, 0.61% of all variants present in both the meta-analysis and eQTL study were eQTL. There were relatively more eQTL detected in the within-population GWAS, with 2.30% of QTL detected for fat percentage, and 3.89% of QTL detected for protein percentage. The percentage of significant variants that were eQTL was higher for the meta-analysis than the GWAS, with 3.38% of variants with a p-value lower than 10^−8^ in the meta-analysis for fat percentage, and 4.60% for protein percentage. The number of eQTL detected using cells collected from milk samples was much lower than that obtained by using blood cells, and only a few (between 0.01% and 0.04%) were among the variants detected in either the GWAS or meta-analysis. All overlaps between eQTL and significant variants were larger than expected by chance. For example, there were 22,152 variants with a p-value ≤ 10^−8^ in the meta-analysis for fat yield, of which nine were eQTL detected from cells collected from milk samples. By chance, the expected number would be only 22,152 $\times$ 10^−6^ = 0.02 eQTL.

**Table 5:** Overlap between eQTL and significant variants

Set	Cells collected from milk samples			Blood cells		
	Total	eQTL	%	Total	eQTL	%
All	9,191,239	6678	0.07	8,587,100	52,802	0.61
p_gwas__fat % ≤ 10^−8^	22,152	9	0.04	20,702	476	2.30
p_meta__fat % ≤ 10^−8^	20,087	3	0.01	18,735	633	3.38
p_gwas__prot % ≤ 10^−8^	28,967	5	0.02	27,781	1081	3.89
p_meta__prot % ≤ 10^−8^	33,911	13	0.04	32,505	1496	4.60

All: all variants present in both meta-analysis and eQTL study; p_gwas__fat % ≤ 10^−8^, p_gwas__prot % ≤ 10^−8^, p_meta__fat % ≤ 10^−8^ and p_meta__prot % ≤ 10^−8^: variants with a p-value ≤ 10^−8^ in at least one of the within population GWAS and meta-analysis for fat and protein percentage, respectively; total: total number of variants in a set of variants, eQTL: number of variants in a set that were eQTL,  %: eQTL total*100%

### Correlations between within-population GWAS

When all variants were used, the effects estimated in the within-populations GWAS were only weakly correlated between populations (see Additional file 7: Tables S3 and S4). Correlations were stronger between GWAS of the same breed. For example, the correlation for effects estimated for protein percentage was 0.15 between HOLF and HOLG, and 0.25 between AUSB and AUSC. Between different breeds, correlations were close to zero. Much higher correlations were observed for significant variants. For example, for fat percentage, the correlation between MON and NOR was 0.03 for all variants, whereas the effects of significant variants had a correlation of 0.72.

### Direction of effect

**Fig. 4:**
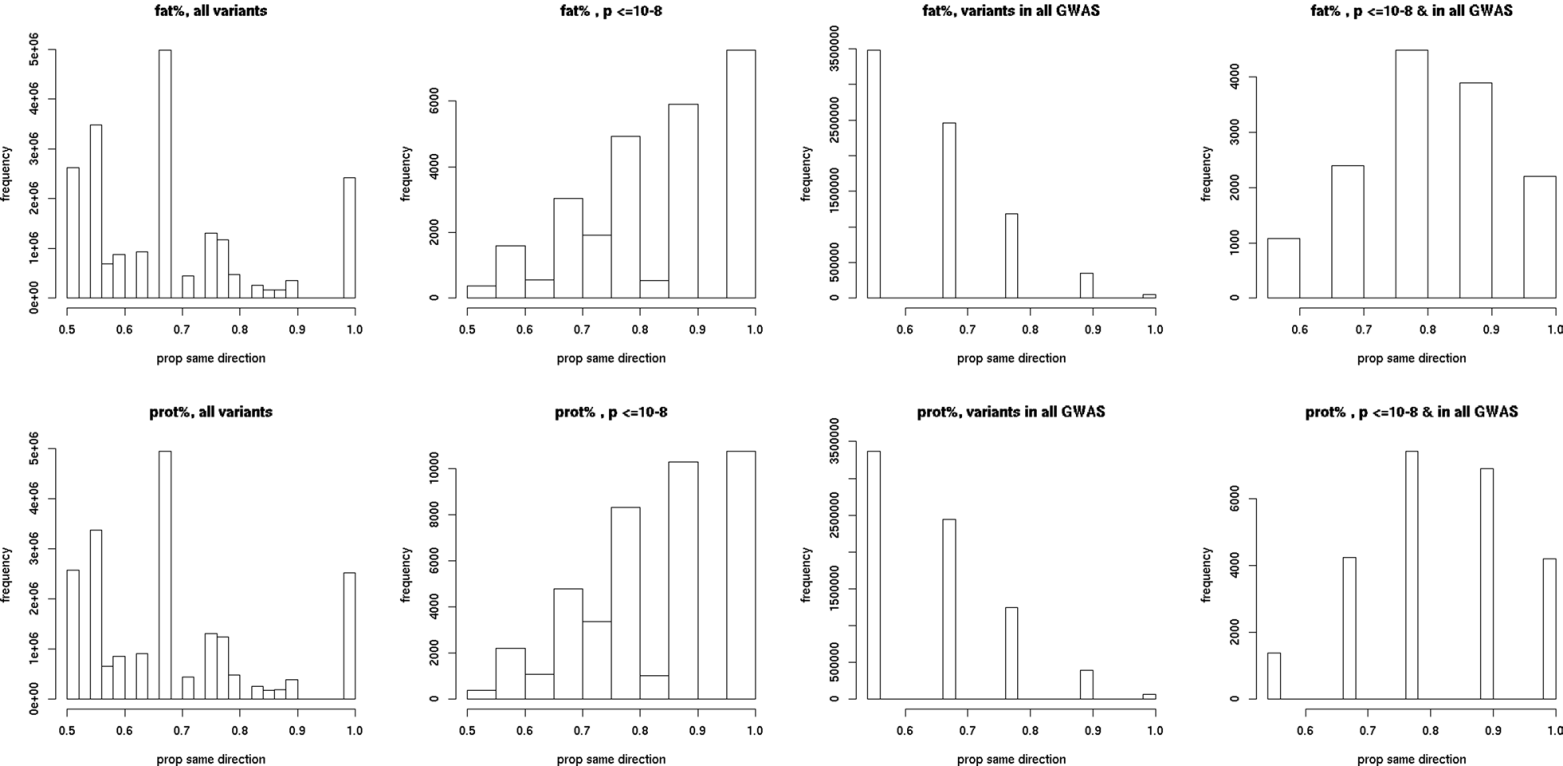
Distribution of the proportion of GWAS with the same direction of effect. From left to right: all variants (all variants), variants with p ≤ 10^−8^ in the meta-analysis (p ≤ 10^−8^), variants included in all GWAS (variants in all GWAS), and variants included in all GWAS and p ≤ 10^−8^ in the meta-analysis (p ≤ 10^−8^ & in all GWAS). Top = fat percentage (fat %), bottom = protein percentage (prot %)

For both fat and protein percentage, significant variants had the same direction of effect in more GWAS than all variants (Fig. 4). For fat percentage, 14, 19, 41 and 26% of significant variants had the same direction of effect in less than four GWAS, four or five GWAS, six or seven GWAS or eight or nine GWAS, respectively, substantially more than the 37, 38, 22 and 2% of all variants. A similar pattern was observed for protein percentage.

The majority of QTL had the same direction of effect in all within-population GWAS that detected them. Of the 138 and 176 lead variants for the QTL detected for fat and protein percentage, respectively 100 and 114 had the same direction of effect in all GWAS in which they were included. Only two variants had significant effects in opposite directions in two populations. Inconsistencies in direction of effect between within-population GWAS that contained Holstein individuals (AUSB, AUSC, HOLF and HOLG) were only observed for four QTL that were detected for fat percentage and two for protein percentage. One of these QTL was a highly significant QTL for both traits, and located at 93,945,991 bp on chromosome 5, in the *MGST1* gene. This QTL had a positive effect on both traits in all GWAS except that for HOLF.

### F_ST_

**Fig. 5:**
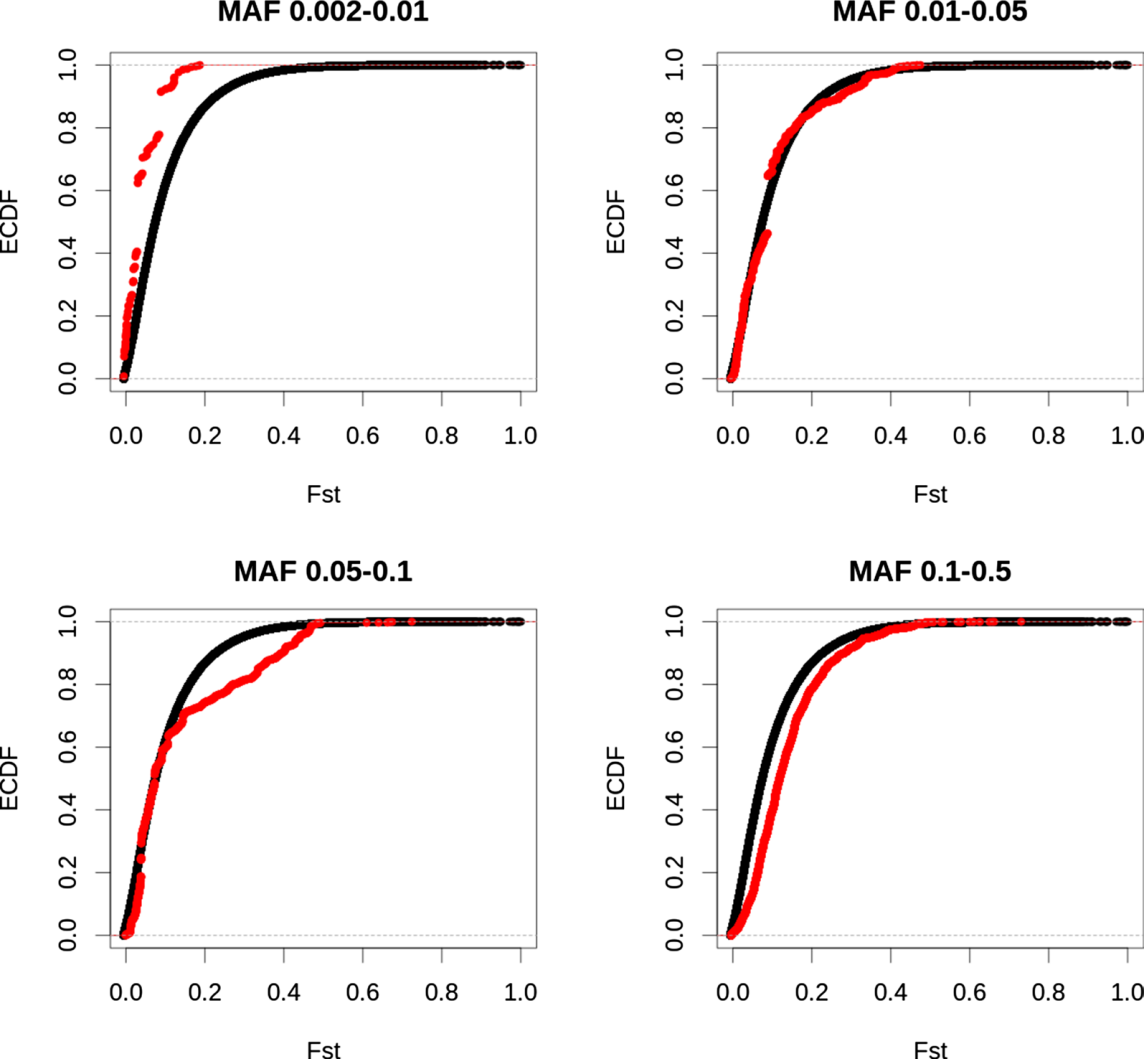
F_ST_ of all sequence variants and significant variants for fat percentage. Empirical cumulative distribution (ECDF) of F_ST_ values of all variants (black) and significant variants (red), for variants with a minor allele frequency between 0.002 and 0.01 (top left), 0.01 and 0.05 (top right), 0.05 and 0.10 (bottom left), and 0.10 and 0.50 (bottom right)

**Fig. 6:**
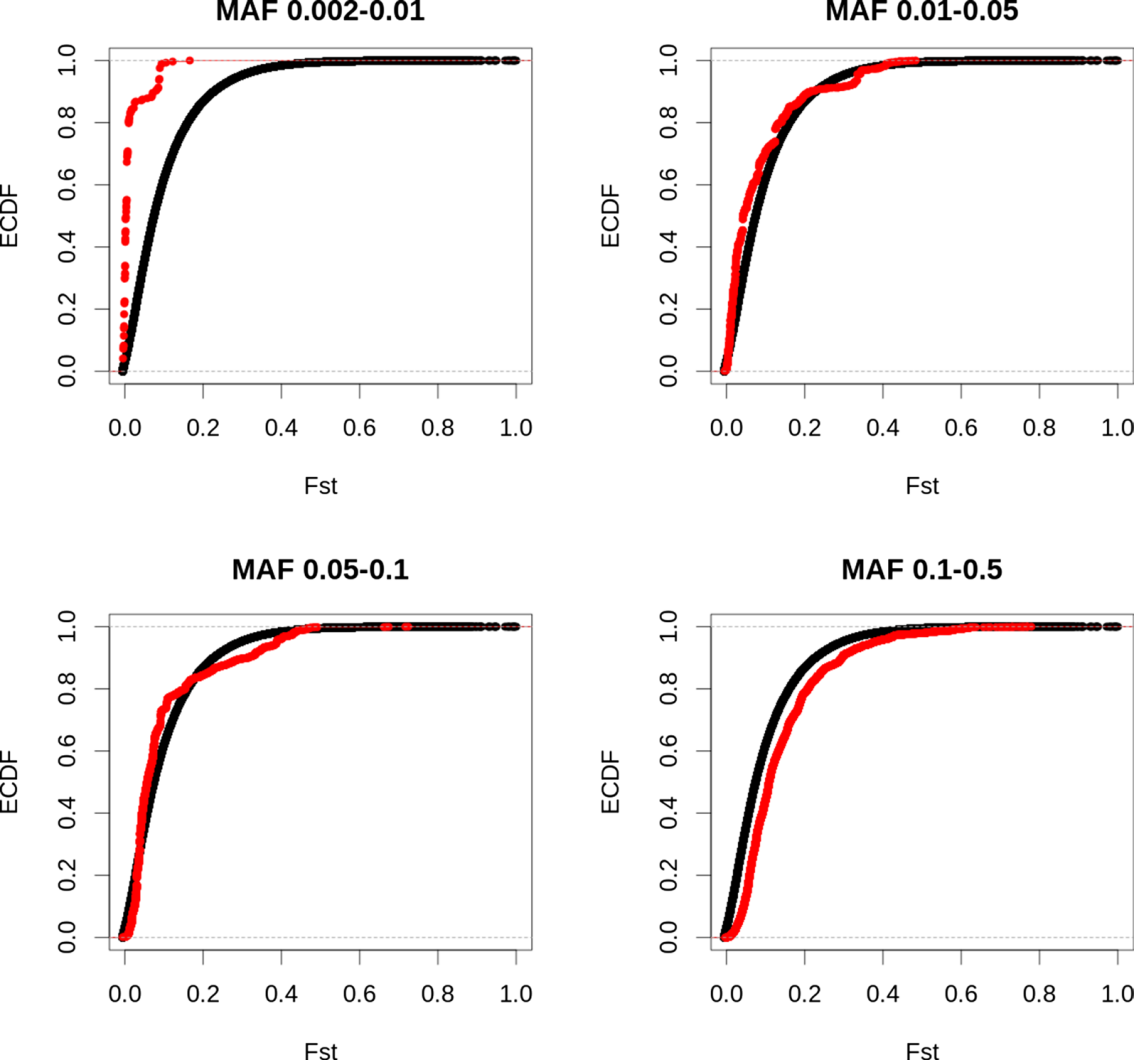
F_ST_ of all sequence variants and significant variants for protein percentage. Empirical cumulative distribution (ECDF) of F_ST_ values of all variants (black) and significant variants (red), for variants with a minor allele frequency between 0.002 and 0.01 (top left), 0.01 and 0.05 (top right), 0.05 and 0.10 (bottom left), and 0.10 and 0.50 (bottom right)

While significant common variants (MAF 0.10–0.50) had slightly larger F_ST_ values than all common variants (Figs. 5 and 6), the opposite was observed for rare variants (MAF 0.002–0.01). Significant rare variants had lower F_ST_ values compared to all rare variants. For variants with a MAF between 0.01 and 0.05, no clear difference in F_ST_ values was observed between significant variants and all variants.

### DAVID analysis

**Fig. 7:**
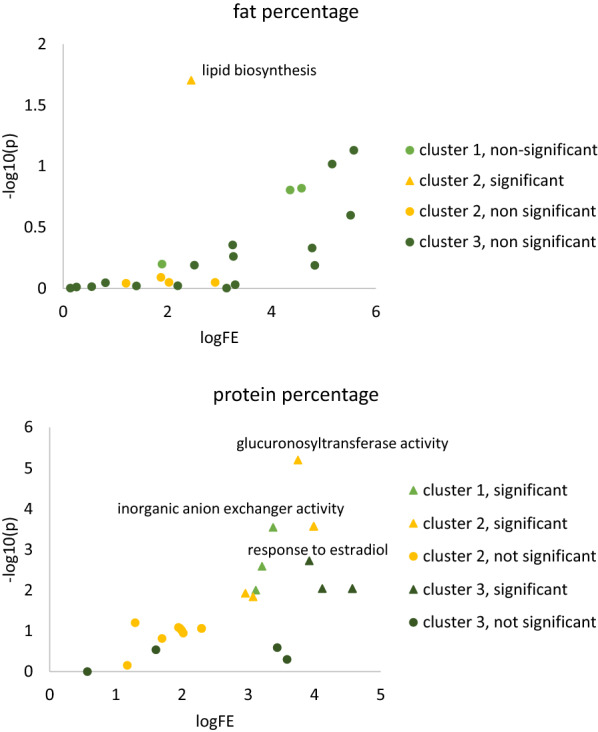
Fold enrichment and significance of keywords in DAVID clusters for fat and protein percentage. Log fold enrichment (logFE) and −log10 p-value after Benjamini–Hochberg correction for multiple testing (−log10(p)) for keywords in the top three clustered detected in DAVID analysis. For cluster, the keyword the top significant variant is annotated on the graph

The DAVID functional annotation tool clustered 80 of 391 genes associated with fat percentage in the meta-analysis and present in the databases used by DAVID in 28 clusters. The enrichment scores of the annotation clusters ranged from 1.98 for the top cluster, to 0.03 for the bottom cluster. Figure 7 shows the fold enrichment and significance (p-value after Benjamini–Hochberg correction for multiple testing [29]) of the keywords in the top three clusters. More details on the clusters are in Additional file 8: Table S5 and Additional file 9: Table S6. The only significant (p ≤ 0.05) keyword associated with fat percentage was the UP_KEYWORD “lipid biosynthesis” in annotation cluster 2, with a p-value of 0.02. The genes associated with this keyword were *MECR*, *FDPS*, *PMVK*, *ST8SIA1*, *PTDSS1*, *HSD17B12*, *PCYT2*, *FASN*, *SCD* and *GPAT4*.

Of the 761 genes associated with protein percentage and present in the database used by DAVID, 118 were clustered in 38 clusters with enrichment scores ranging from 5.18 to 0. The first cluster includes several solute carrier (SLC) genes, significantly (p-value ≤ 0.05 after Benjamini–Hochberg correction for multiple testing [29]) associated with GOTERM “inorganic anion exchanger activity”. The second cluster contained several genes that are significantly associated with GOTERM_MF_DIRECT “glucuronosyltransferase activity”, GOTERM_BP_DIRECT “flavonoid biosynthetic process” and “flavonoid glucuronidation” and KEGG_PATHWAYs “pentose and glucuronate interconversions” and “ascorbate and aldarate metabolism”. The third cluster contained several genes that encode milk proteins, including *LALBA* on chromosome 5, and *CSN2*, *CSN1S2, CSN1S1* and *CSN3* on chromosome 6, and are significantly associated with GOTERM_BP_DIRECT “response to oestradiol”, “response to 11-deoxycorticosterone” and “response to “dehydroepiandrosterone” and UP_KEYWORD “milk protein”.

## Discussion

Our meta-analyses detected more QTL with fewer significant variants per QTL and at lower FDR than the within-population GWAS, which suggests that across-population meta-analysis of summary statistics both increases power and mapping precision compared to within-population GWAS. A multi breed meta-analysis can increase both power and precision because on the one hand, the sample size and thus power increase, and on the other hand, including data from multiple breeds breaks down LD, which can reduce the number of variants associated with a QTL and thereby increase mapping precision [6]. The number of QTL detected in our meta-analysis was substantially larger than that detected by Pausch et al. [7], while similar detection criteria were used, which indicates that the more than five-fold larger sample in our study enabled us to detect more QTL.

By setting a distance of at least 1 Mb between adjacent QTL, our aim was to reduce the number of detected QTL that were associated with the same true QTL. However, LD may be conserved over more than 1 Mb, and consequently, very significant QTL such as *DGAT1* on chromosome 14 can have variants that are associated with them over a longer distance. Therefore, we performed a COJO analysis to test how many of the detected QTL were independent of each other. Because we were not able to combine the original datasets used for the meta-analysis, we used an alternative dataset that contained sequences representing most of the populations included in the meta-analysis to estimate the LD structure in the COJO analysis. While the small size of this dataset may have reduced the accuracy of the COJO analysis, this dataset was more representative of the LD structure across the populations used in the meta-analysis than a larger within-population dataset. The COJO analysis reduced the number of QTL, which indicates that the numbers of QTL reported are inflated. The number of independent QTL retained by COJO (74 for fat percentage and 84 for protein percentage) remained more than double the number of QTL detected by Pausch et al. [7].

Not all of the QTL detected in the within-population GWAS were detected in the meta-analysis. Because of the smaller data size of the within-population GWAS, the number of false positives detected by the within-population GWAS may be larger than in the meta-analysis, and some of the QTL detected within-population but not in the meta-analysis may be false positives. However, there can be several reasons why a true QTL is significant in a within-population GWAS but not significant in a meta-analysis. One reason could be that a SNP is significantly associated with a trait in one population because it is in LD with the causal variant in that population. However, the LD between this SNP and the causal variant might differ in other populations and thus results in a weakened association between SNP and trait. Ideally, the causal variant would be detected as the most significant local variant, but this does not always occur due to sampling error and, in some cases, the causal variant is not included in the data, at least not for all populations. Furthermore, a variant in high LD with the causal variant could be more significant than the causal variant if it has a higher imputation accuracy than the causal variant. Another reason why a QTL may be detected in a within-population GWAS but not in the meta-analysis could be that multiple causal variants with opposing effects are present in the same region. Different breeds may have different causal mutations segregating in the same region, which can reduce significance in the meta-analysis. Epistasis may be another factor why a within-breed QTL is not detected in the meta-analysis, since it can cause some QTL to have opposing effects in different breeds [30]. We suggest that this reversal of effect of causal variants is rare because we only observed two variants with an effect that was significant in two populations but opposite in sign.

To validate the QTL that were detected in the meta-analysis, we performed a validation meta-analysis. All QTL detected for protein percentage and 87% of the QTL detected for fat percentage had the same direction of effect in the validation as in the original meta-analysis, whereas by chance alone, only 50% would have the same direction. Most QTL were not significant in the validation meta-analysis, which is not surprising, given the much smaller size of the validation dataset and the fact that it included only cows, which have a less accurate “phenotype” than bulls. This is also consistent with our previous observation, i.e. that the comparison of the directions of variant effects across GWAS results is more powerful than the comparison of p-values across GWAS [12]. As previously stated, different LD structures in different GWAS populations can lead to different selections of top variants using the same p-value threshold. Nevertheless, we still found that more than 12% of the QTL detected for fat and protein percentage from the discovery analysis were significant in the validation analysis. This amount of overlap is more than that expected by chance.

### Comparison with known QTL and causal mutations

We detected QTL that encompass genes that are well known for their effect on production traits in dairy cattle, such as *MGST1* on chromosome 5 [31], *ABCG2* on chromosome 6 [32], *PAEP* on chromosome 11 [33], *DGAT1* on chromosome 14 [34], and *GHR* on chromosome 20 [35]. For a few of these QTL, the underlying causal mutations are known. While we detected QTL near the causal variants, the most significant variant in our study was not always the causal variant. For example, the causal variant for *DGAT1* [34] is located at 1,802,265-1,802,266 bp on chromosome 14. In the meta-analysis, the variant at 1,802,266 bp was highly significant with a p-value of 1.3 × 10^−996^ for fat percentage. However, it was not the most significant variant in the meta-analysis, most likely because it was filtered out from the HOLG data because of low imputation accuracy. Similarly, for the QTL located near *ABCG2* on chromosome 6, the most significant variant with a p-value of 3.4 × 10^−106^ for protein percentage was located at 38,031,954 bp and was more significant than the causal variant [32] at 38,027,010 bp (p = 5.6 × 10^−92^) because the causal variant was not included in the AUSB dataset. These examples demonstrate that the most significant variant in GWAS or meta-analysis is not necessarily the known causal variant. In contrast, the causal variant for *GHR* [35] was the most significant variant in the meta-analysis for both fat and protein percentage. While the absence of the causal variant in some GWAS may explain why another variant is more significant in the meta-analysis than the causal variant, it is also possible that there are multiple causal variants present in the same region. However, LD of a segregating variant with multiple causal variants can result in the highest significance.

Jiang et al. [36] reported a GWAS on USA Holstein cattle based on sequence variants in or near genes. Our meta-analysis detected a QTL within 1 Mb of the candidate variants reported for the 12 QTL detected for fat percentage and 23 QTL detected for protein percentage in US Holstein bulls by Jiang et al. [36]. The lead variant was the same for eight of these QTL (for fat percentage: 93,945,738 bp on chromosome 5 and 38,027,010 bp on chromosome 6, and for protein percentage: 31,349,638 bp on chromosome 5, 38,027,010 bp and 87,154,594 bp on chromosome 6, 1,801,116 on chromosome 14 and 9,563,396 on chromosome 29). Almost all the candidate variants listed by Jiang et al. [36] were significant in the meta-analysis. Exceptions were one QTL for fat percentage located at 74,829,183 bp on chromosome 15, that had a p-value of 1.5 × 10^−5^ in the meta-analysis, and three QTL for protein percentage located on chromosome 1, 14 and 21 that had p-values of 6.6 × 10^−8^, 3.8 × 10^−7^ and 2.7 × 10^−8^, respectively, in the meta-analysis. Because Jiang et al. [36] preselected sequence variants and, in that process, excluded intronic and intergenic variants, the majority of the QTL detected in the meta-analysis were not included in their study. Their GWAS did contain multiple variants within 1 Mb of each of our QTL, and there was at least one variant with a p-value ≤ 10^−8^ in the GWAS by Jiang et al. [36] for 27 of the QTL detected in the meta-analysis for fat percentage and 59 for protein percentage. For 21 of the QTL detected for fat percentage and 50 of the QTL for protein percentage, the top variant in the meta-analysis was included in the GWAS of Jiang et al. [36]. Among these, eight QTL for fat percentage and 10 QTL for protein percentage were significant in their study. In the GWAS of Jiang et al. [36], the lack of significance of most of the QTL detected in the meta-analysis may be attributed to the larger sample size and inclusion of multiple breeds in our analysis. In another study, Marete et al. [9] reported QTL detected for fat and protein percentage in a large meta-analysis using data from French Holstein, Montbéliarde and Normande cattle. The overlap with QTL detected by Marete et al. [9] and our meta-analysis was smaller than that with Jiang et al. [36], with only 20 of 48 and 6 of 29 QTL detected by for fat yield and percentage and protein yield and percentage, respectively. While the SNP genotypes used by Marete et al. [9] contained several candidate causal mutations, it was not a sequence-based GWAS, which may explain the small overlap between their results and this study. The dataset used in the meta-analysis by Pausch et al. [7] was used in our analyses, and all QTL detected by Pausch et al. [7] were confirmed in our analyses, except one QTL detected for protein percentage at 56,528,040 bp on chromosome 4, that had a p-value of 2.3 × 10^−7^ in our meta-analysis.

### Minor allele frequency

While, in agreement with previous studies in cattle [37], sequence variants were enriched for low-frequency MAF classes, the majority of QTL that we detected had moderate to high MAF. This is in line with Pausch et al. [7], who detected only few QTL with a MAF lower than 0.05. The lack of rare QTL does not mean that most QTL in dairy cattle are common variants, but more likely it indicates that, in spite of the large sample size, our study had a relatively low statistical power to detect QTL with low MAF and a lower imputation accuracy for low MAF QTL than for more common QTL.

If the effect of a QTL is independent of the MAF (p), then the power to detect the QTL is proportional to p(1−p). However, the number of variants with MAF = p is proportional to 1/[p(1−p)]. Thus, one might expect the number of significant variants to be independent of MAF. Alternatively, if the variance explained by a QTL is independent of MAF, the number of significant variants should increase sharply at low MAF just as the number of all variants does. In fact, our results are in between these two alternatives, which suggests that the size of the effect of QTL increases as MAF declines but not sufficiently to prevent the explained variance (and the power to detect) from decreasing. This is the same conclusion as Zeng et al. [38].

The meta-analysis appeared to favour variants with a high MAF in Holstein (see Additional file 5: Figure S3 and Additional file 6: Figure S4), which was the breed origin of most of the animals included in the meta-analysis. Using the same MAF threshold in the meta-analysis and the within-population GWAS may have influenced the number of QTL detected in each analysis. The applied MAF filter corresponds with a minor allele count (MAC) of 377 for the meta-analysis, but to a much lower MAC within-population (between 6.5 and 129). This has likely contributed to the smaller number of QTL detected in the within-population GWAS and illustrates the advantage of a meta-analysis over within-population GWAS.

### F_ST_

We observed slightly higher F_ST_ values for significant common variants compared to all variants, but lower F_ST_ values for significant rare variants. Pausch et al. [7] also observed higher F_ST_ values for QTL than for non-QTL. The slightly higher F_ST_ values for significant variants compared to all variants may indicate different selection pressures in different breeds. For instance, mutations that increase fat percentage by decreasing milk volume might be selected for in some breeds, but selected against in other breeds. While selection on milk composition has been much more limited than on milk yield, a correlated response is still expected due to the correlation between milk yield and composition. In our study, high F_ST_ values at some variants may indicate differences in selection pressure between the breeds. Kemper et al. [39] found no convincing evidence of selection for several major QTL detected for production traits in dairy cattle, and Xiang et al. [40] showed only a small contribution of variants under selection to various quantitative traits in dairy cattle. Further research is required to identify potential links between F_ST_ value, allele frequency and significance.

### Functional annotations

The proportion of genic variants, located in a gene or in the upstream or downstream region of a gene, was higher among the significant variants of the meta-analysis than among all variants. This is in agreement with the depletion of intergenic effects and enrichment of genic classes for variants associated with various traits in dairy cattle reported in other studies [41, 42]. Interestingly, a large part of the variants with significant effects was located in non-coding regions that may have a regulatory function, such as intronic, upstream and downstream regions.

The DAVID analysis identified several clusters enriched for certain functional annotations. Some of the clusters could be described as milk protein genes (e.g. *LALBA*), or anion exchanges and solute carrier genes (e.g. *SLC*). Whereas some of the genes in these classes, *LALBA* [43] on chromosome 5 and the casein genes on chromosome 6 [44] are genes known to be associated with major QTL in dairy cattle, other genes were not and these clusters may provide novel candidate genes for milk and fat percentage. For example, the second cluster for fat percentage contained 10 genes associated with lipid biosynthesis: *MECR* on chromosome 2, *FDPS* and *PMVK* on chromosome 3, *ST8SIA1* on chromosome 5, *PTDSS1* on chromosome 14, *HSD17B12* on chromosome 15, *PCYT2* and *FASN* on chromosome 19, SCD on chromosome 26 and *GPAT4* on chromosome 27. Some of these genes, including *MECR* [45], *FASN* [46], *SCD* [47] and *GPAT4* [48], have previously been identified as candidate genes for milk traits in dairy cattle.

Our data and analysis were based on the previous UMD3.1 bovine reference genome because the more recent ARS-UCD1.2 reference was not available at the time the within-population GWAS were performed. The same meta-analysis, carried out using the ARS-UCD1.2 reference, may lead to a better targeting of causative mutations [49]. Except for one top variant associated with a QTL detected for protein percentage, all top QTL variants were present on the ARS-UCD1.2 reference.

### Overlap between significant variants and eQTL

The depletion or enrichment of significant variants for eQTL was not consistent across tissues. While significant variants were enriched for eQTL when blood cells were used to detect eQTL, using cells collected from milk samples resulted in significant variants being depleted for eQTL. This is in line with the eQTL study, in which the power to detect significant variants was higher using blood cells than using cells collected from milk samples [23]. Interestingly, whereas the original eQTL study found only little overlap between eQTL and QTL, even with blood cells, we found some enrichment of QTL for blood eQTL, which is potentially due to the higher power of the meta-analysis compared to the data used in the original study [21]. Other studies have shown some evidence of overlap between QTL and eQTL. Littlejohn et al. [31] used mammary tissue to detect an eQTL at the *MGST1* gene that is also a well-known QTL for milk traits in dairy cattle, and Xiang et al. [25] showed overlap between eQTL and QTL for several traits in dairy cattle in milk and blood cells.

## Conclusions

Our study identified a large number of QTL that are associated with fat and protein percentage in dairy cattle. We confirmed the efficiency of a large-scale multi-breed meta-analysis and studied the properties of significant variants compared to all variants. Significant variants are more often common variants, which indicates that either most QTL have a high MAF, or that even with the large sample size used in our study, we still have insufficient power to identify and fine map rare QTL. The percentage of intergenic variants was substantially lower for significant variants than for non-significant variants. A large part of the significant variants was located in non-coding, potentially regulatory regions. In some cases, the genes near the QTL shared a common function such as genes involved in lipid synthesis affecting fat percentage. Except for rare variants, significant variants tend to have higher F_ST_ than all variants.

## Supplementary information

**Additional file 1: Figure S1.** Manhattan plots of within-population GWAS for fat and protein percentage. The red line indicates p = 10^−8^.

**Additional file 2: Table S1.** QTL detected for fat percentage in the meta-analysis. chr = chromosome, pos = position in base pair on UMD3.1 assembly, ARS-UCD2.1 = position in base pair on ASR-UCD2.1 assembly, p = p-value in the meta-analysis, dir = direction of effect in each of the GWAS (from left to right: Braunvieh, Fleckvieh, German Holstein, Norwegian Red, Australian bull dataset, Australian cow dataset, Montbéliarde, Normande, French Holstein), start = pos – 250 kb, end = pos + 250 kb, nSig = number of variants with a p-value ≤ 10^−8^ in the interval, nGenic = nSig associated with a gene, genes = genes in the interval with significant variants, cojo = p-value in COJO analysis for retained variants, or discarded/not present to indicate variants that were discarded or not included in COJO analysis), sameDirVal = indicates whether a variant had the same direction of Z-score in the meta-analysis and the validation analysis (- = not included in validation analysis, 0 = opposite direction, 1 = same direction), pVal = p-value in the validation analysis.

**Additional file 3: Table S2.** QTL detected for protein percentage in the meta-analysis. chr = chromosome, pos = position in base pair on UMD3.1 assembly, ARS-UCD2.1 = position in base pair on ASR-UCD2.1 assembly, p = p-value in the meta-analysis, dir = direction of effect in each of the GWAS (from left to right: Braunvieh, Fleckvieh, German Holstein, Norwegian Red, Australian bull dataset, Australian cow dataset, Montbéliarde, Normande, French Holstein), start = pos – 250 Kb, end = pos + 250 Kb, nSig = number of variants with a p-value ≤ 10^−8^ in the interval, nGenic = nSig associated with a gene, genes = genes in the interval with significant variants, cojo = p-value in COJO analysis for retained variants, or discarded/not present to indicate variants that were discarded or not included in COJO analysis), sameDirVal = indicates whether a variant had the same direction of Z-score in the meta-analysis and the validation analysis (- = not included in validation analysis, 0 = opposite direction, 1 = same direction), pVal = p-value in the validation analysis.

**Additional file 4: Figure S2.** QTL detected on chromosome 25. Association of variants around 36 Mb on chromosome 25 with protein percentage in the meta-analysis (top) and GWAS for Norwegian Red (bottom).

**Additional file 5: Figure S3.** Distribution of within-population minor allele frequencies (MAF) of all variants and significant variants. Significant variants had a p-value ≤ 10^−8^ in the meta-analysis.

**Additional file 6: Figure S4.** Distribution of minor allele frequencies within-population (MAF) of all variants and significant variants. Significant variants had a p-value ≤ 10^−8^ in the meta-analysis.

**Additional file 7: Table S3 and Table S4.** Correlations between effects estimated in within population GWAS. Tables S3 and S4 show the results for fat percentage and protein percentage, respectively; above the diagonal = all variants, below the diagonal = significant variants (p ≤ 10^−8^). AUSB = Australian bull dataset, AUSC = Australian cow dataset, HOLF = French Holstein, MON = Montbéliarde, NOR = Normande, NR = Norwegian Red, HOLG = German Holstein, BRAU = Braunvieh, FLCK = Fleckvieh.

**Additional file 8: Table S5.** DAVID analysis for fat percentage. Three clusters with the highest enrichment scores according to DAVID functional annotation clustering for genes associated with significant variants in the meta-analysis for fat percentage.

**Additional file 9: Table S6.** DAVID analysis for protein percentage. Three clusters with the highest enrichment scores according to DAVID functional annotation clustering for genes associated with significant variants in the meta-analysis for protein percentage.

## Data Availability

The data used for this research is not publicly available.
