# Supplementary material for: Meta-analysis for milk fat and protein percentage using imputed sequence variant genotypes in 94,321 cattle from eight cattle breeds
Source: Genet Sel Evol. 2020 Jul 7;52:37. doi: 10.1186/s12711-020-00556-4 (PMC7339598; doi:10.1186/s12711-020-00556-4)

**Australian bulls, fat percentage**


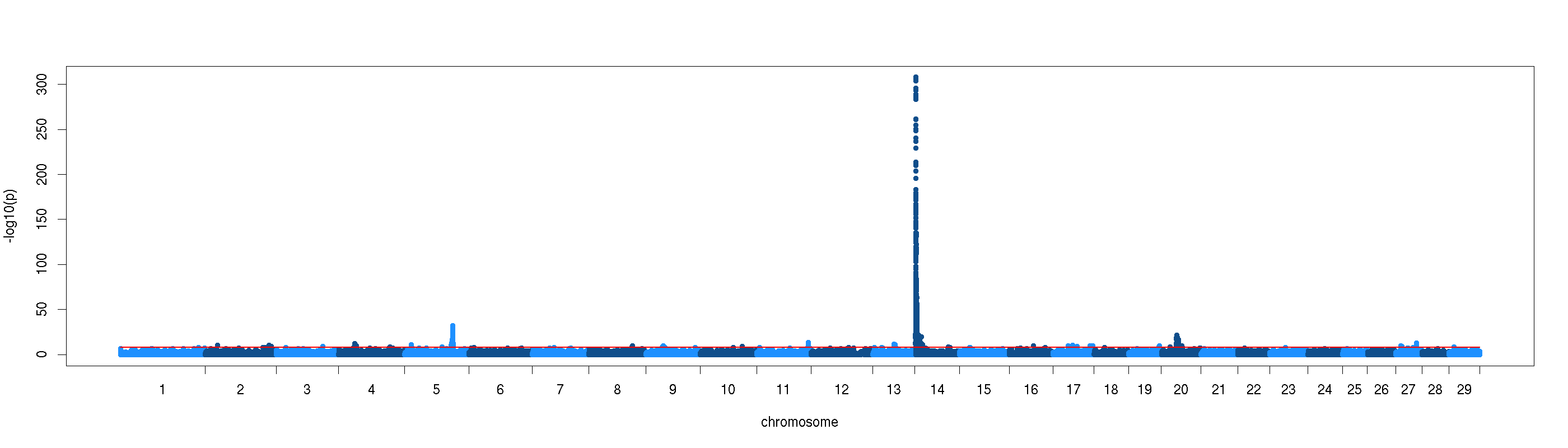


**Australian cows, fat percentage**


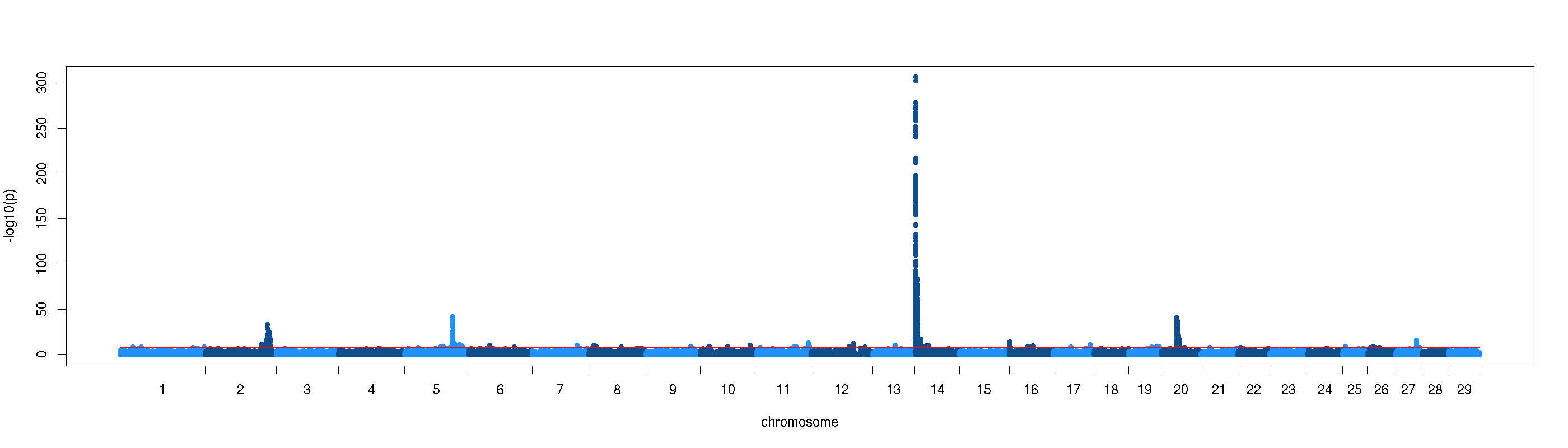


**French Holstein, fat percentage**


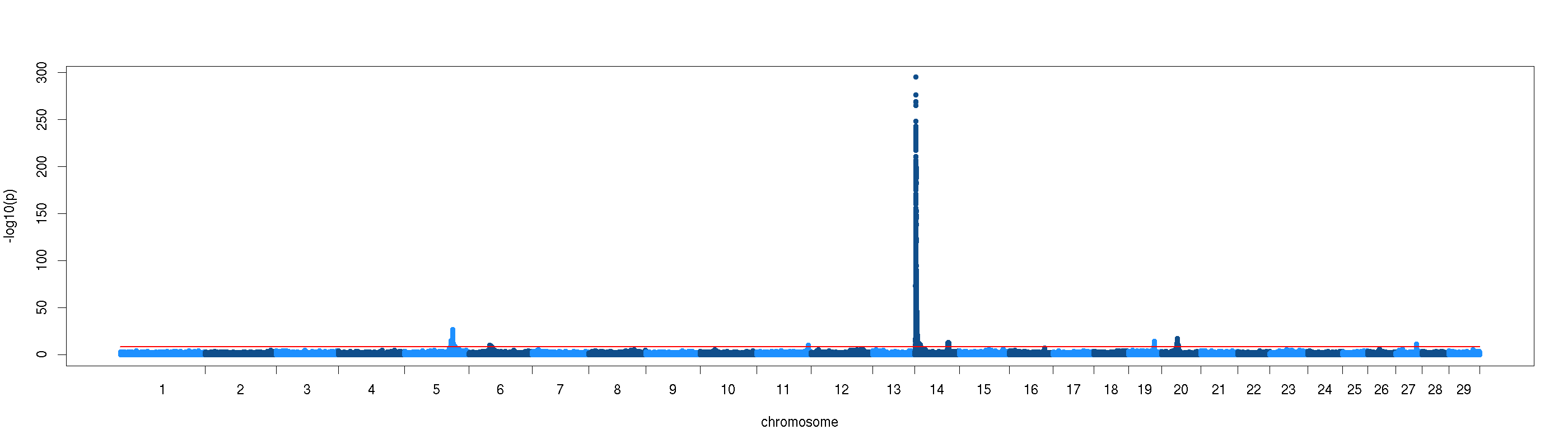


**Montbéliarde, fat percentage**


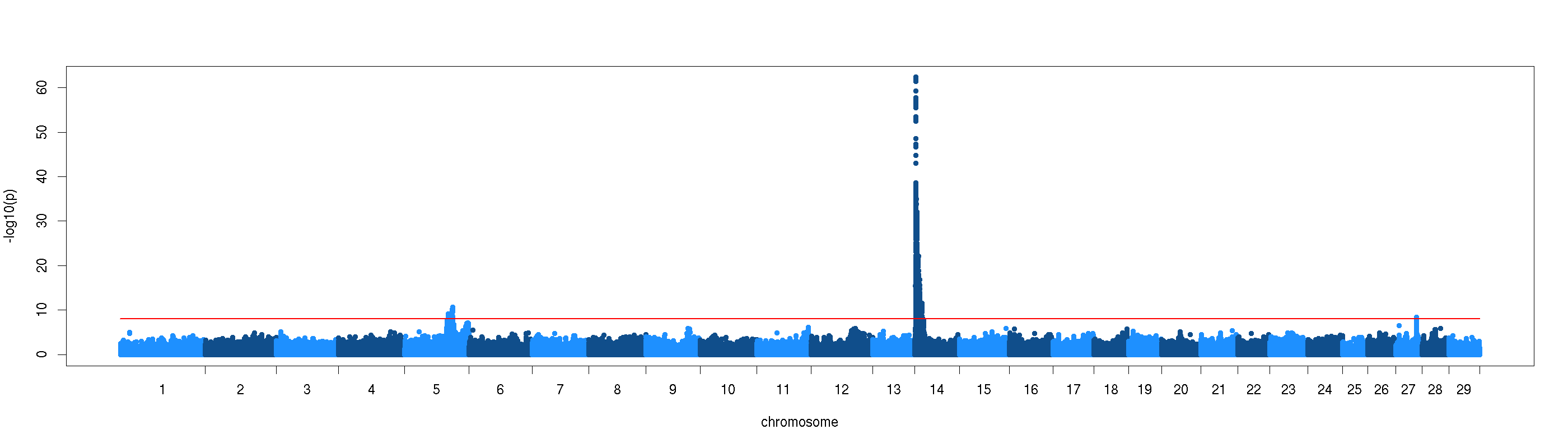


**Normande, fat percentage**


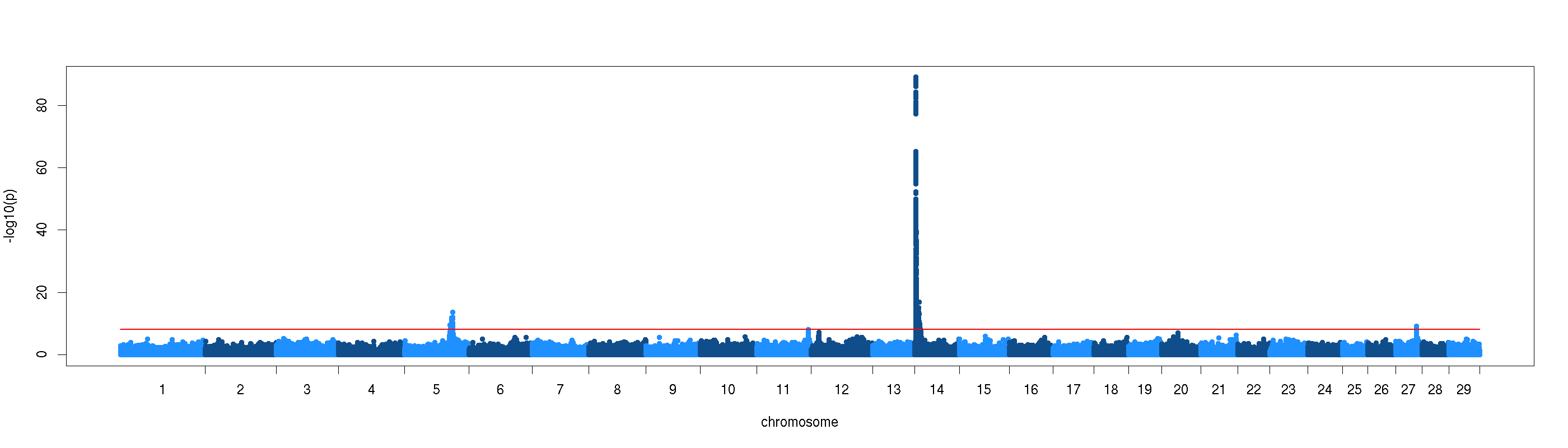


**Norwegian Red, fat percentage**


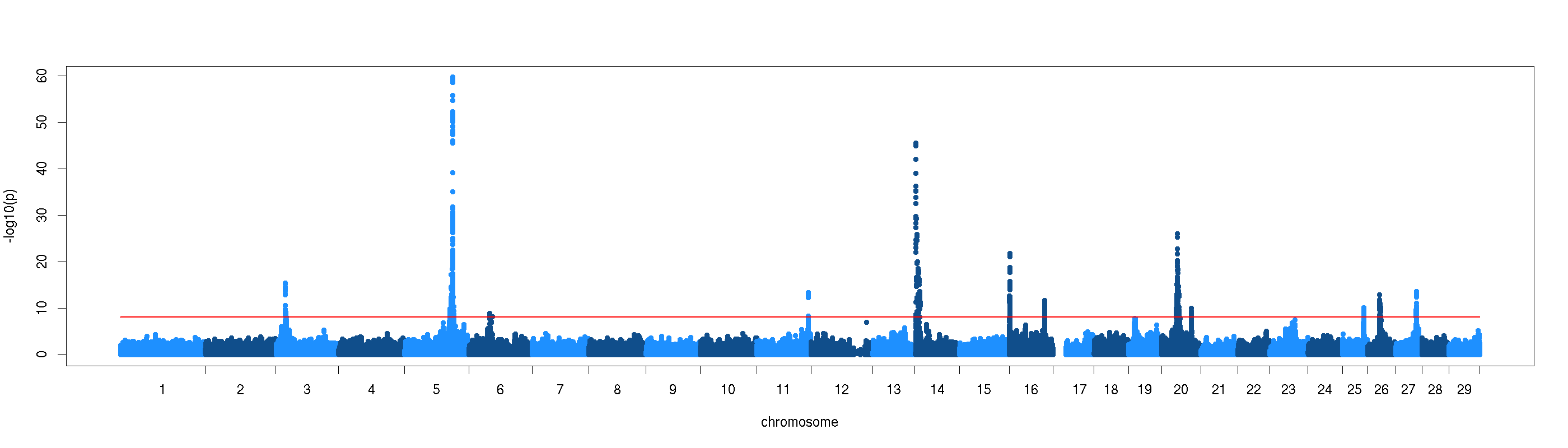


**German Holstein, fat percentage**


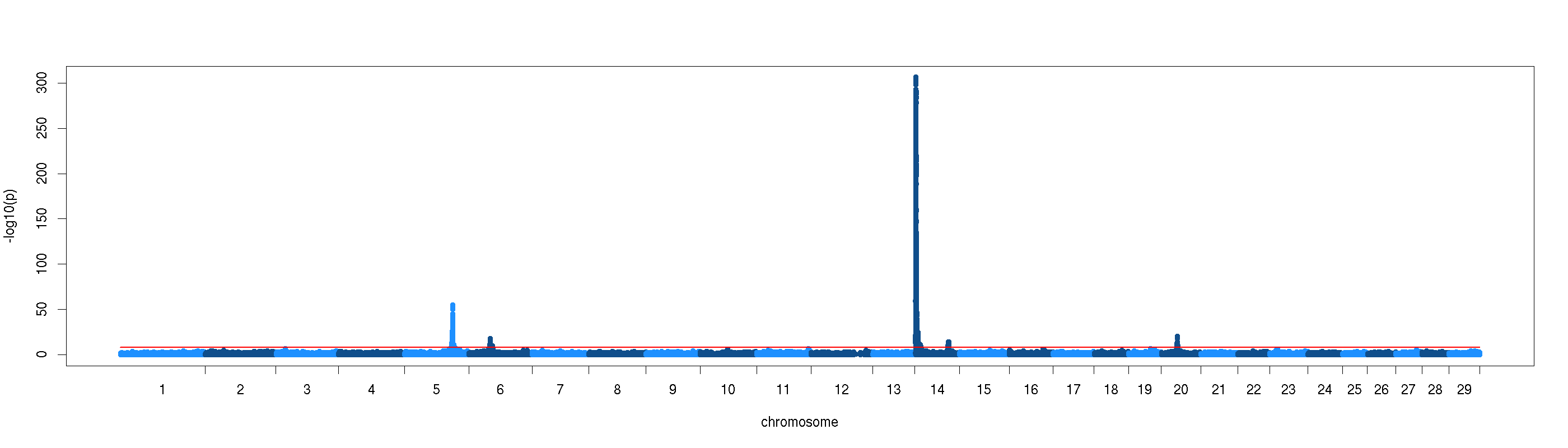


**Braunvieh, fat percentage**


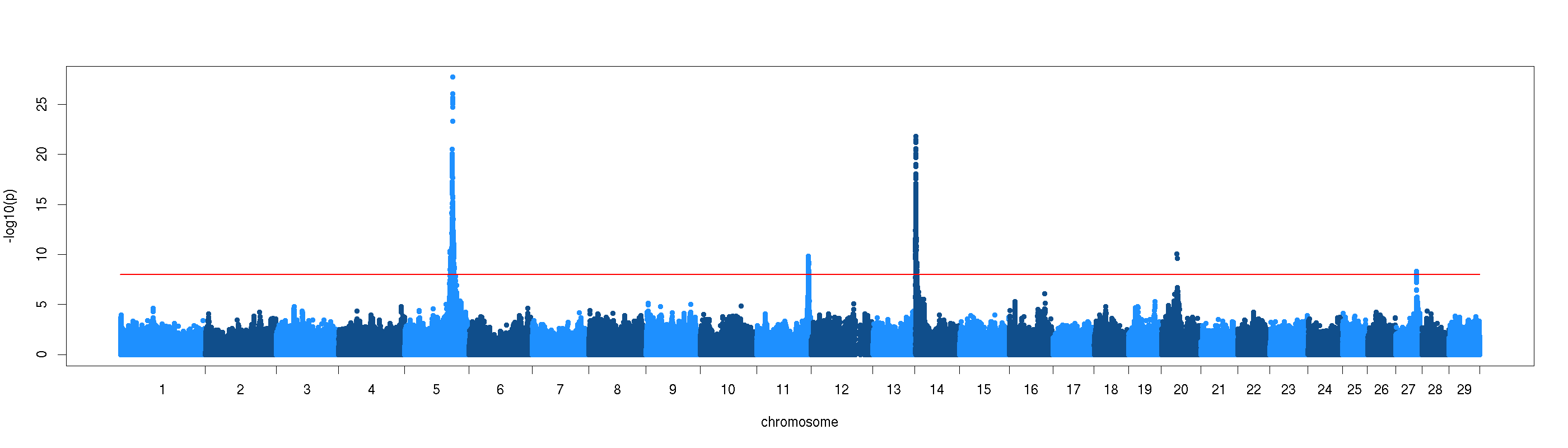


**Fleckvieh, fat percentage**


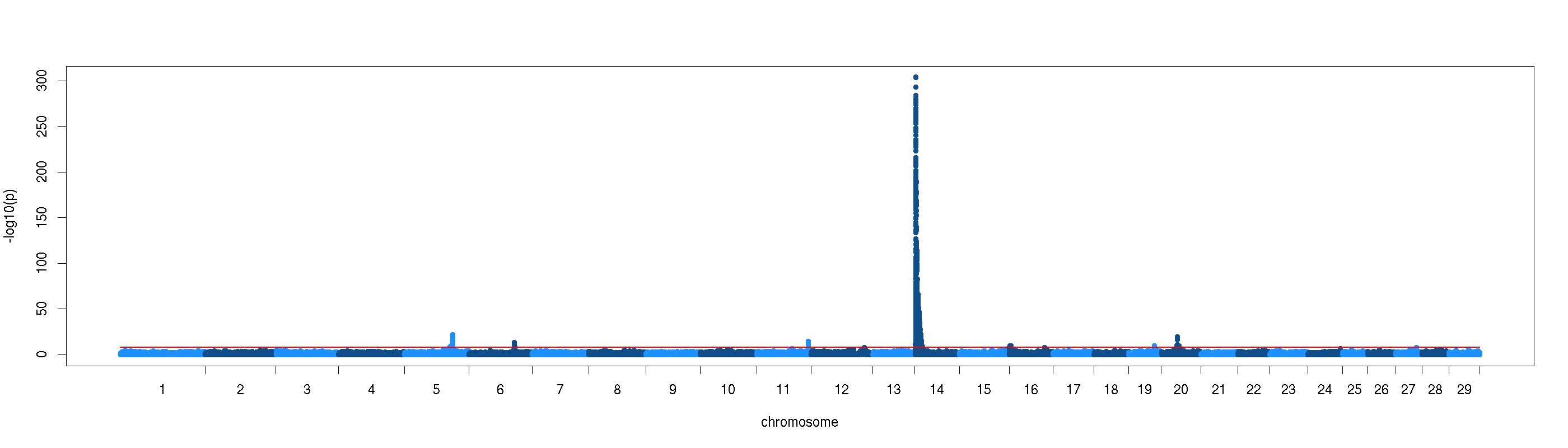


**Australian bulls, protein percentage**


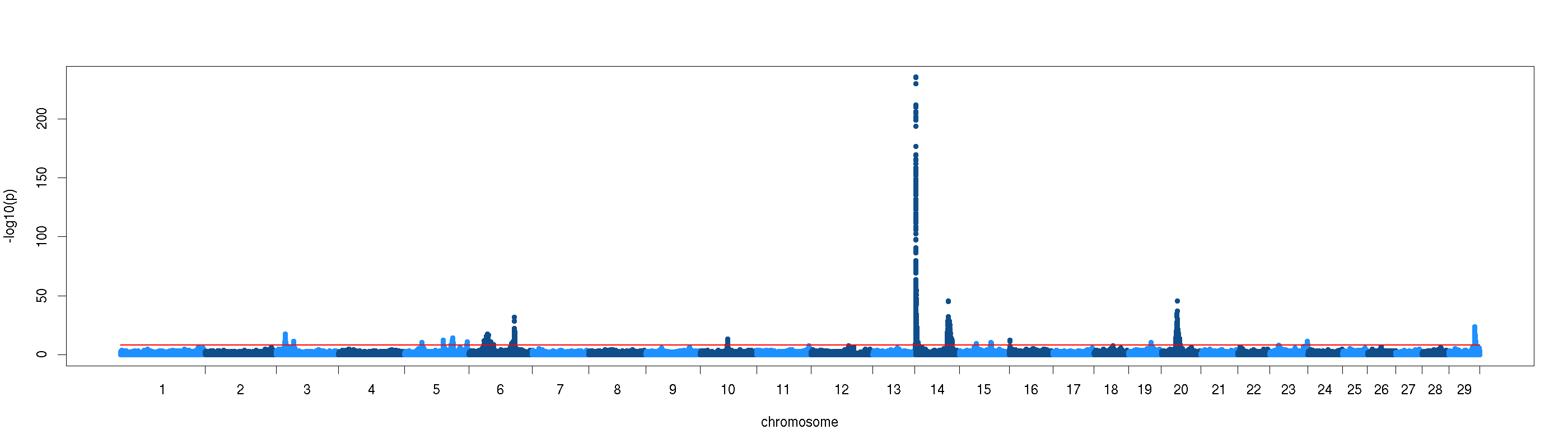


**Australian cows, protein percentage**


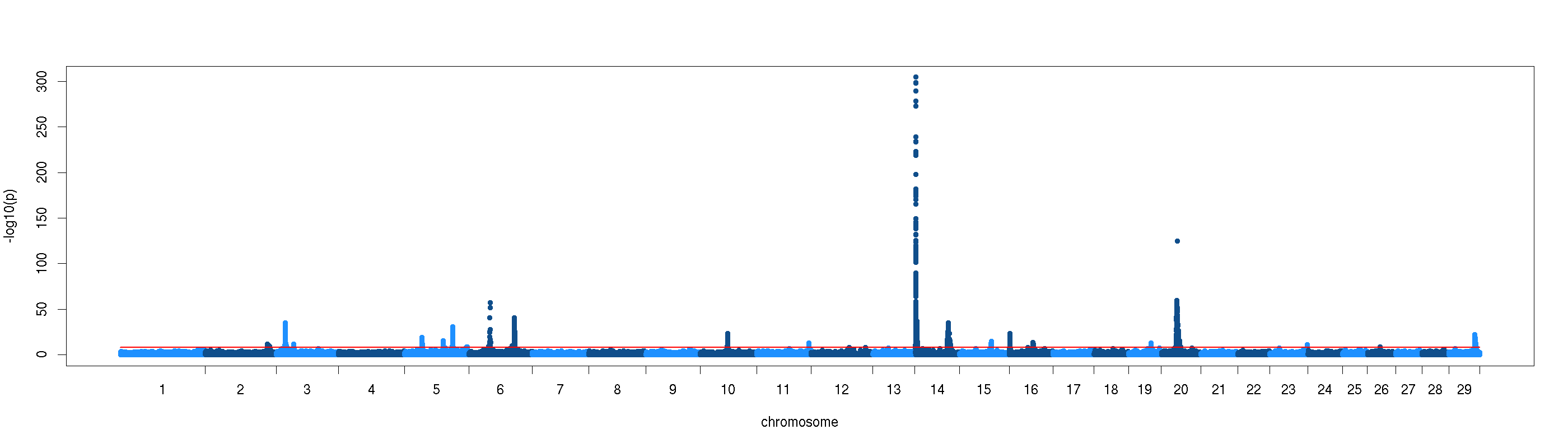


**French Holstein, protein percentage**


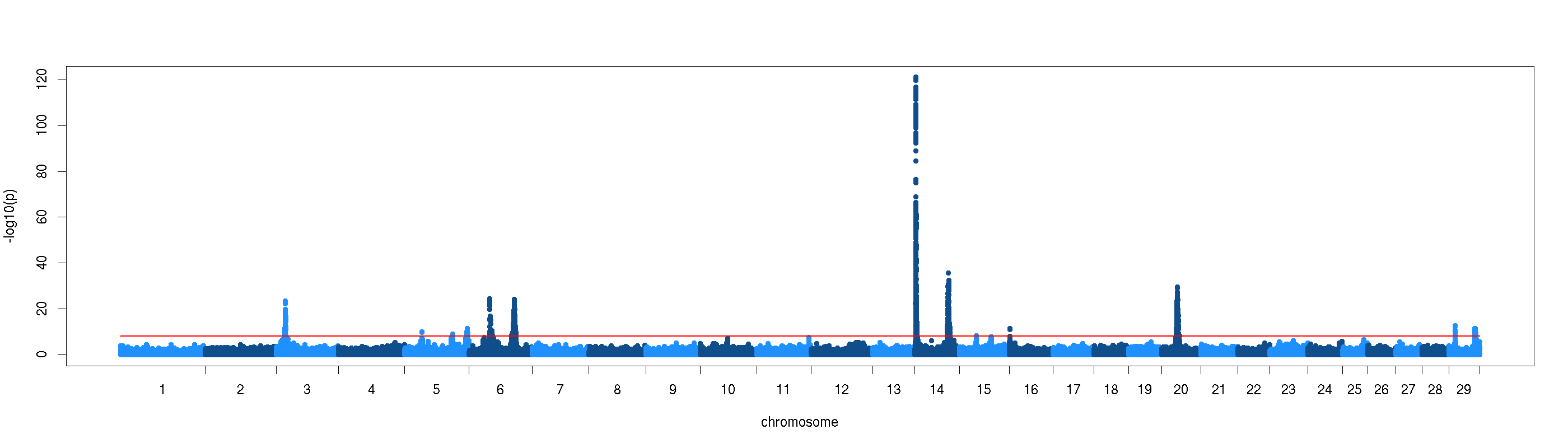


**Montbéliarde, protein percentage**


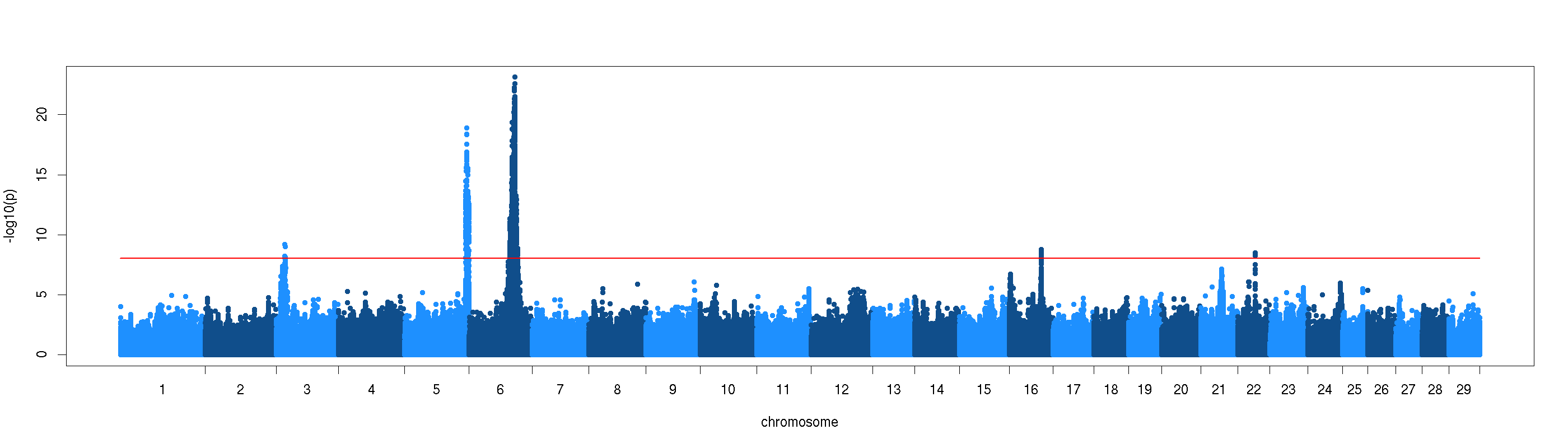


**Normande, protein percentage**


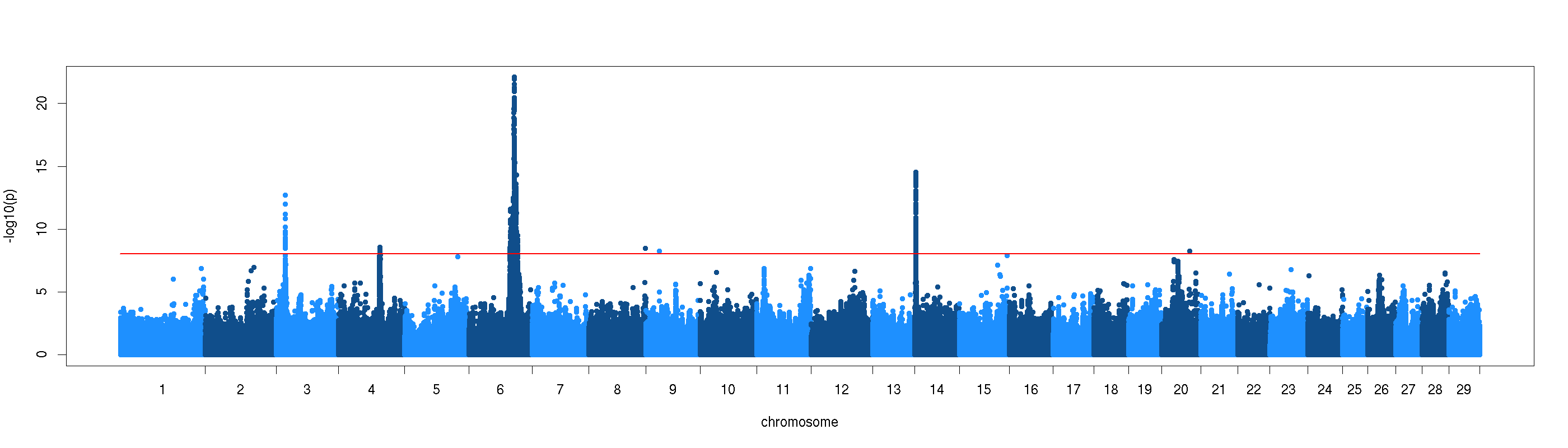


**Norwegian Red, protein percentage**


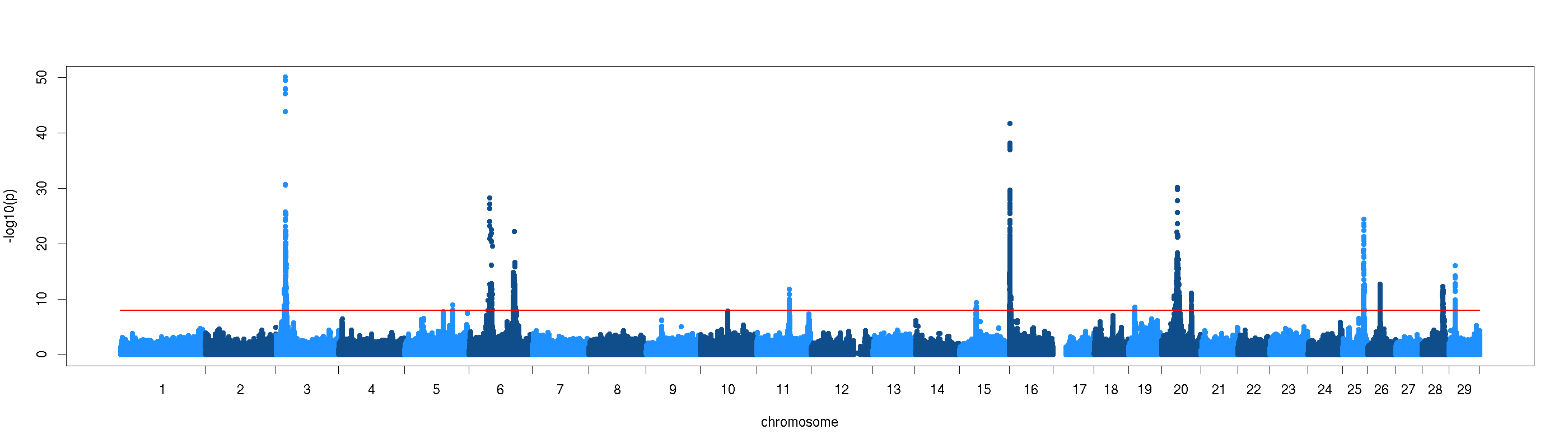


**German Holstein, protein percentage**


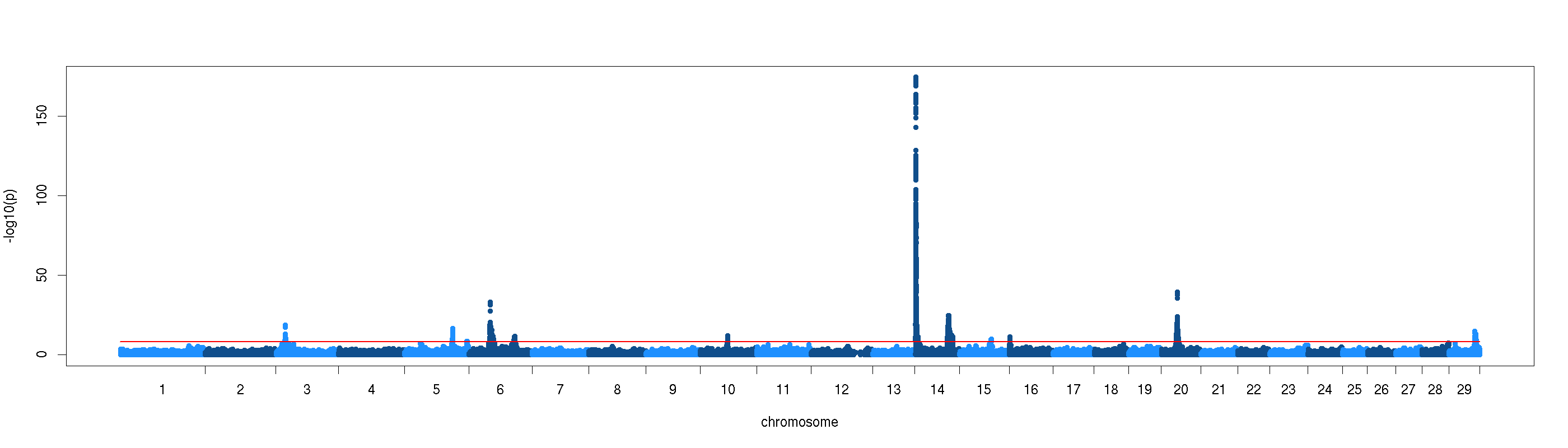


**Braunvieh, protein percentage**


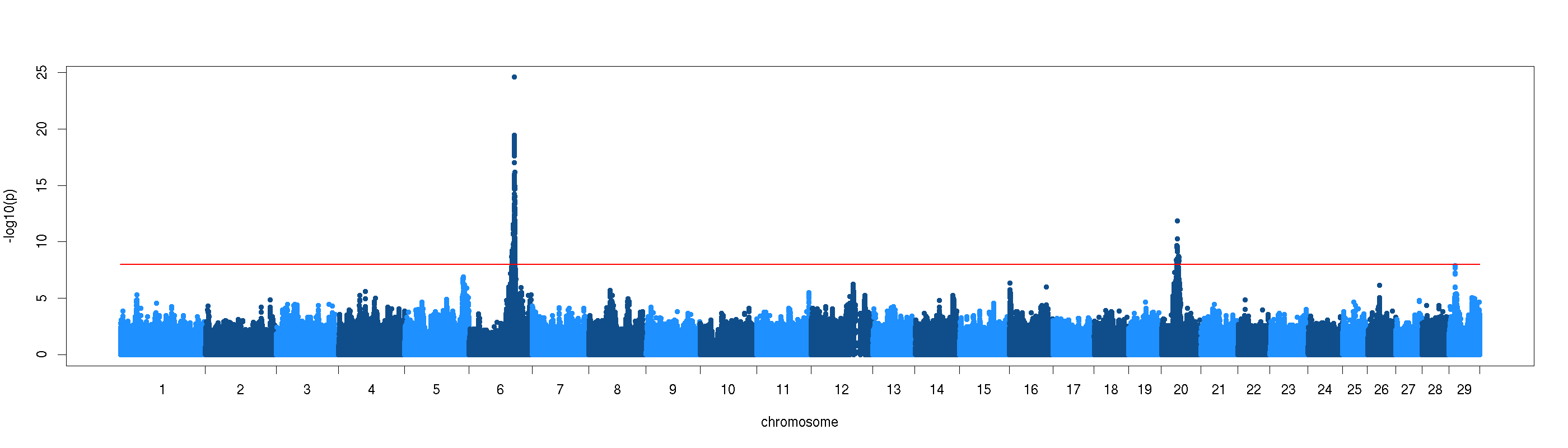


**Fleckvieh, protein percentage**


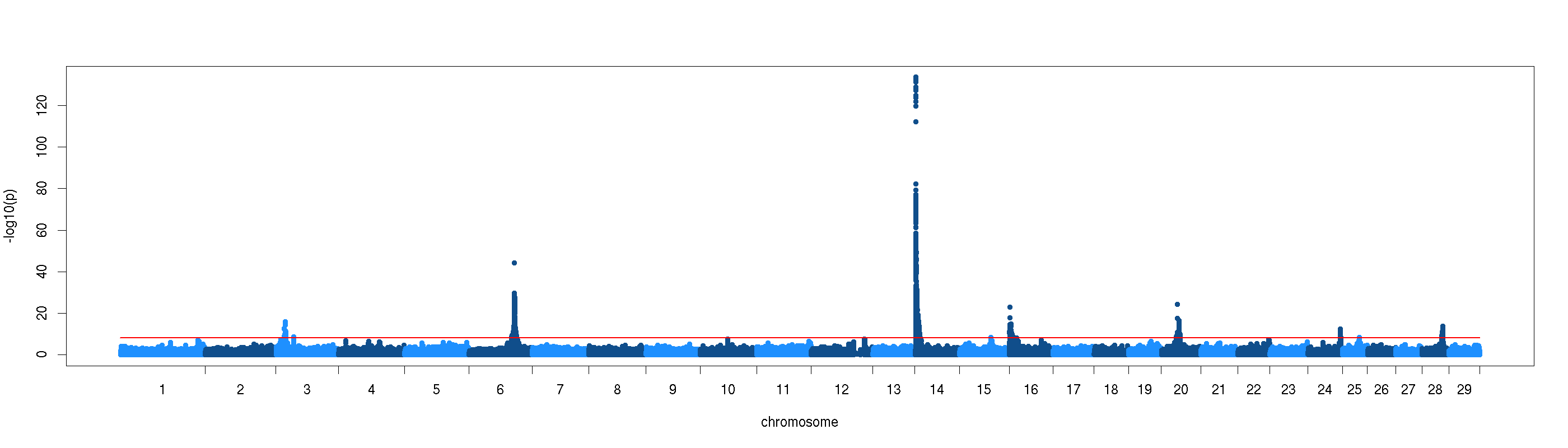

Supplement: Supplementary file 1 — Additional file 1: Figure S1. Manhattan plots of within-population GWAS for fat and protein percentage. The red line indicates p = 10−8. [file 12711_2020_556_MOESM1_ESM.docx]
