# Supplementary figures and images for: Meta-analysis for milk fat and protein percentage using imputed sequence variant genotypes in 94,321 cattle from eight cattle breeds
Source: Genet Sel Evol. 2020 Jul 7;52:37. doi: 10.1186/s12711-020-00556-4 (PMC7339598; doi:10.1186/s12711-020-00556-4)

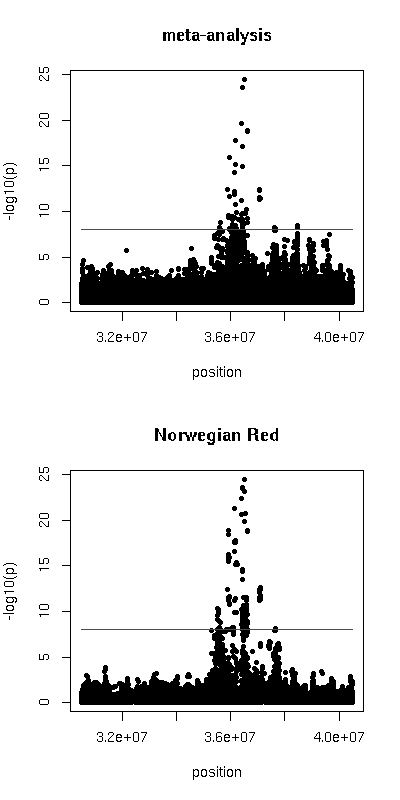

Supplement: Supplementary file 4 — Additional file 4: Figure S2. QTL detected on chromosome 25. Association of variants around 36 Mb on chromosome 25 with protein percentage in the meta-analysis (top) and GWAS for Norwegian Red (bottom). [file 12711_2020_556_MOESM4_ESM.png]

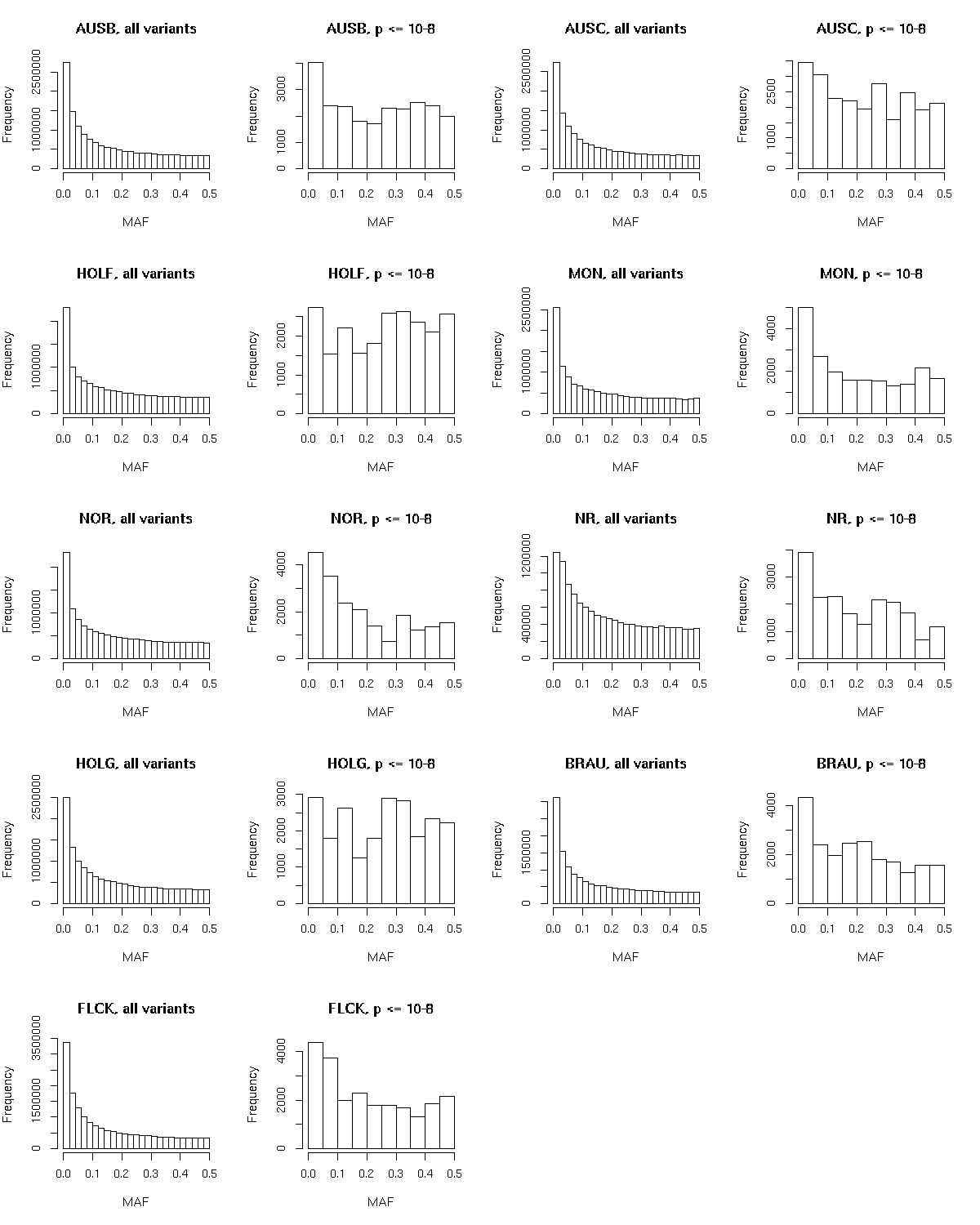

Supplement: Supplementary file 5 — Additional file 5: Figure S3. Distribution of within-population minor allele frequencies (MAF) of all variants and significant variants. Significant variants had a p-value ≤ 10−8 in the meta-analysis. [file 12711_2020_556_MOESM5_ESM.png]

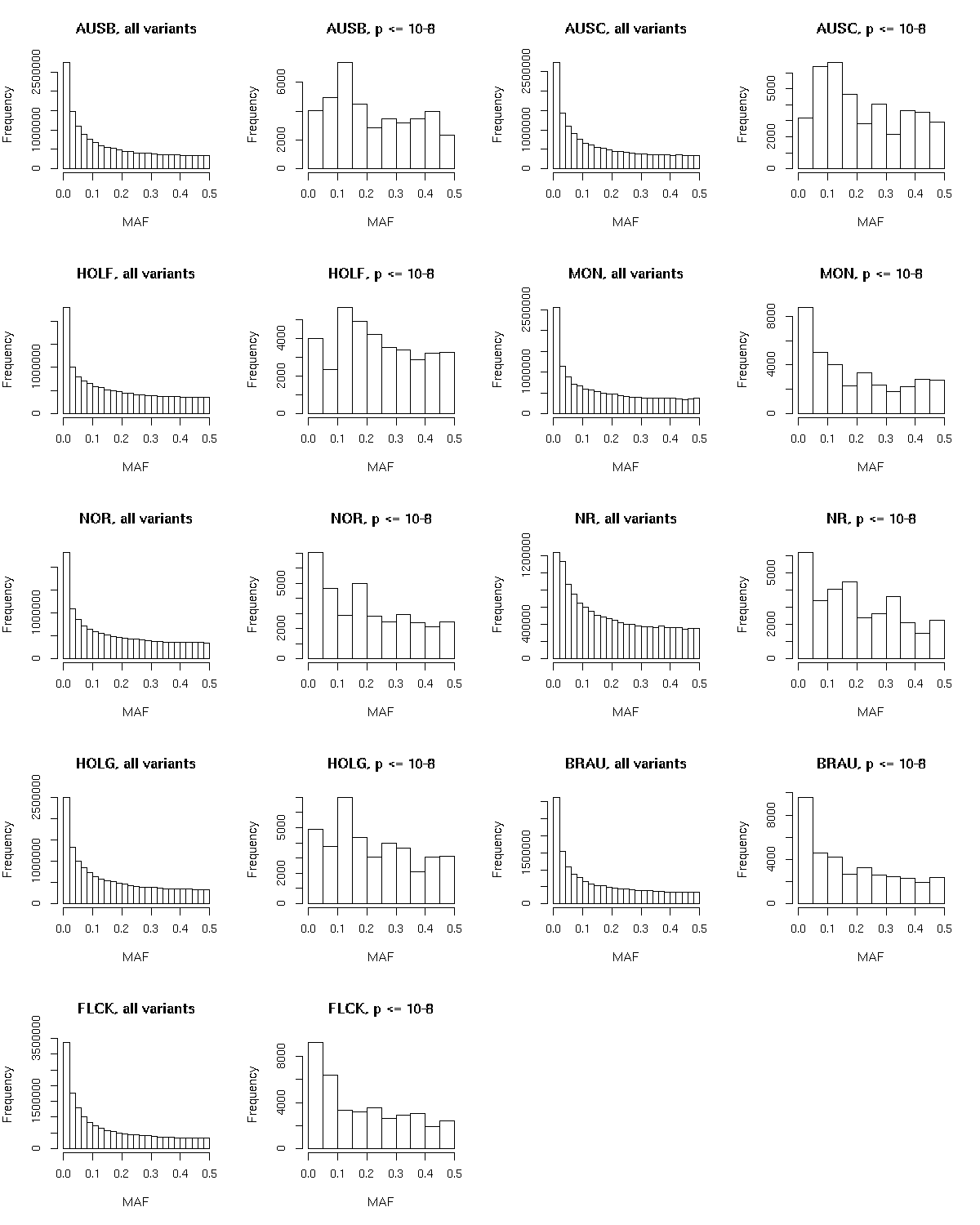

Supplement: Supplementary file 6 — Additional file 6: Figure S4. Distribution of minor allele frequencies within-population (MAF) of all variants and significant variants. Significant variants had a p-value ≤ 10−8 in the meta-analysis. [file 12711_2020_556_MOESM6_ESM.png]
