# Supplementary material for: Meta-analysis for milk fat and protein percentage using imputed sequence variant genotypes in 94,321 cattle from eight cattle breeds
Source: Genet Sel Evol. 2020 Jul 7;52:37. doi: 10.1186/s12711-020-00556-4 (PMC7339598; doi:10.1186/s12711-020-00556-4)
Supplement: Supplementary file 7 — Additional file 7: Table S3 and Table S4. Correlations between effects estimated in within population GWAS. Tables S3 and S4 show the results for fat percentage and protein percentage, respectively; above the diagonal = all variants, below the diagonal = significant variants (p ≤ 10−8). AUSB = Australian bull dataset, AUSC = Australian cow dataset, HOLF = French Holstein, MON = Montbéliarde, NOR = Normande, NR = Norwegian Red, HOLG = German Holstein, BRAU = Braunvieh, FLCK = Fleckvieh. [file 12711_2020_556_MOESM7_ESM.docx]

**Table 1. Correlations between effects estimated in within-population GWAS for fat percentage.**

| **pop** | **AUSB** | **AUSC** | **HOLF** | **MON** | **NOR** | **NR** | **HOLG** | **BRAU** | **FLCK** |
| --- | --- | --- | --- | --- | --- | --- | --- | --- | --- |
| AUSB |  | 0.20 | 0.10 | 0.02 | 0.01 | 0.04 | 0.15 | 0.01 | 0.02 |
| AUSC | 0.97 |  | 0.08 | 0.02 | 0.00 | 0.03 | 0.12 | 0.00 | 0.03 |
| HOLF | 0.67 | 0.67 |  | 0.03 | 0.02 | 0.05 | 0.15 | 0.00 | 0.04 |
| MON | 0.58 | 0.57 | 0.55 |  | 0.01 | 0.03 | 0.03 | 0.00 | 0.05 |
| NOR | 0.21 | 0.26 | 0.28 | 0.89 |  | 0.02 | 0.01 | 0.00 | 0.02 |
| NR | 0.92 | 0.88 | 0.83 | 0.72 | 0.90 |  | 0.05 | 0.01 | 0.06 |
| HOLG | 0.96 | 0.90 | 0.68 | 0.53 | 0.32 | 0.91 |  | 0.01 | 0.05 |
| BRAU | 0.59 | 0.54 | 0.40 | 0.53 | 0.39 | 0.96 | 0.62 |  | 0.01 |
| FLCK | 0.78 | 0.70 | 0.58 | 0.59 | 0.76 | 0.93 | 0.72 | 0.50 |  |

Above diagonals = all variants, below diagonals = significant variants (p ≤ 10^-8^). AUSB = Australian bull dataset, AUSC = Australian cow dataset, HOLF = French Holstein, MON = Montbéliarde, NOR = Normande, NR = Norwegian Red, HOLG = German Holstein, BRAU = Braunvieh, FLCK = Fleckvieh

**Table 2. Correlations between effects estimated in within-population GWAS for protein percentage.**  Above diagonals = all variants, below diagonals = significant variants (p ≤ 10^-8^)

| **pop** | **AUSB** | **AUSC** | **HOLF** | **MON** | **NOR** | **NR** | **HOLG** | **BRAU** | **FLCK** |
| --- | --- | --- | --- | --- | --- | --- | --- | --- | --- |
| AUSB |  | 0.25 | 0.15 | 0.01 | 0.02 | 0.06 | 0.21 | 0.01 | 0.04 |
| AUSC | 0.98 |  | 0.12 | 0.01 | 0.01 | 0.07 | 0.17 | 0.01 | 0.04 |
| HOLF | 0.71 | 0.73 |  | 0.02 | 0.02 | 0.05 | 0.15 | 0.00 | 0.03 |
| MON | 0.72 | 0.88 | 0.83 |  | 0.02 | 0.01 | 0.01 | 0.00 | 0.03 |
| NOR | 0.37 | 0.39 | 0.64 | 0.77 |  | 0.02 | 0.01 | 0.00 | 0.01 |
| NR | 0.89 | 0.89 | 0.80 | 0.93 | 0.67 |  | 0.06 | 0.01 | 0.04 |
| HOLG | 0.98 | 0.95 | 0.68 | 0.93 | 0.31 | 0.93 |  | 0.00 | 0.05 |
| BRAU | 0.46 | 0.73 | -0.54 | 0.31 | 0.15 | -0.12 | 0.87 |  | 0.01 |
| FLCK | 0.81 | 0.87 | 0.64 | 0.68 | 0.67 | 0.96 | 0.82 | 0.80 |  |

Above diagonals = all variants, below diagonals = significant variants (p ≤ 10^-8^). AUSB = Australian bull dataset, AUSC = Australian cow dataset, HOLF = French Holstein, MON = Montbéliarde, NOR = Normande, NR = Norwegian Red, HOLG = German Holstein, BRAU = Braunvieh, FLCK = Fleckvieh
